# Uptake, Metabolism**,** and Accumulation of Tire Wear Particle-Derived Compounds in Lettuce

**DOI:** 10.1021/acs.est.2c05660

**Published:** 2022-12-28

**Authors:** Stephanie Castan, Anya Sherman, Ruoting Peng, Michael T. Zumstein, Wolfgang Wanek, Thorsten Hüffer, Thilo Hofmann

**Affiliations:** † Centre for Microbiology and Environmental Systems Science, Environmental Geosciences EDGE, University of Vienna, 1090Vienna, Austria; ‡ Doctoral School in Microbiology and Environmental Science, University of Vienna, 1090Vienna, Austria; § Research Platform for Plastics in the Environment and Society (PLENTY), University of Vienna, 1090Vienna, Austria; ∥ Centre for Microbiology and Environmental Systems Science, Division of Terrestrial Ecosystem Research, University of Vienna, 1030Vienna, Austria

**Keywords:** tire additives, microplastics, plant uptake, 6PPD, HMMM, PMOC, HRMS, contaminant exposure

## Abstract

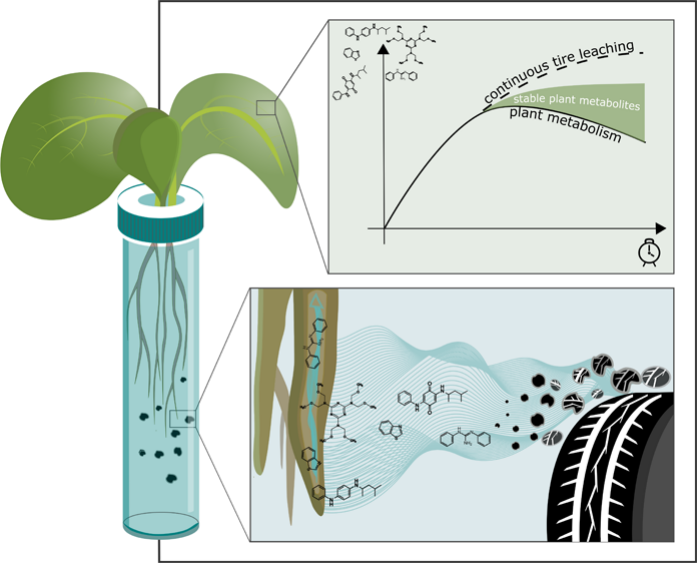

Tire wear particle (TWP)-derived compounds may be of high concern to consumers when released in the root zone of edible plants. We exposed lettuce plants to the TWP-derived compounds diphenylguanidine (DPG), hexamethoxymethylmelamine (HMMM), benzothiazole (BTZ), *N*-phenyl-N′-(1,3-dimethylbutyl)-*p*-phenylenediamine (6PPD), and its quinone transformation product (6PPD-q) at concentrations of 1 mg L^–1^ in hydroponic solutions over 14 days to analyze if they are taken up and metabolized by the plants. Assuming that TWP may be a long-term source of TWP-derived compounds to plants, we further investigated the effect of leaching from TWP on the concentration of leachate compounds in lettuce leaves by adding constantly leaching TWP to the hydroponic solutions. Concentrations in leaves, roots, and nutrient solution were quantified by triple quadrupole mass spectrometry, and metabolites in the leaves were identified by Orbitrap high resolution mass spectrometry. This study demonstrates that TWP-derived compounds are readily taken up by lettuce with measured maximum leaf concentrations between ∼0.75 (6PPD) and 20 μg g^–1^ (HMMM). Although these compounds were metabolized in the plant, we identified several transformation products, most of which proved to be more stable in the lettuce leaves than the parent compounds. Furthermore, continuous leaching from TWP led to a resupply and replenishment of the metabolized compounds in the lettuce leaves. The stability of metabolized TWP-derived compounds with largely unknown toxicities is particularly concerning and is an important new aspect for the impact assessment of TWP in the environment.

## Abstract 2

Edible plants readily take up TWP-derived compounds. Although the compounds are metabolized in the plant, they are replenished by continuous tire leaching and their transformation products accumulate in the leaves.

## 1 Introduction

The extent of continuous tire wear particle (TWP) emissions into the environment remains poorly quantified,^1^ but world-wide emissions are estimated to amount to 5.9 million tons per year.^2^ TWP are expected to be introduced to farmland soils via several pathways, including atmospheric deposition and road runoff. Additionally, high retention of TWP in wastewater treatment plants (WWTP), with an estimated amount of >93% of TWP retained, implicates that WWTP sludge is an important source of TWP to farmlands when biosolids are applied as fertilizers.^3^ It was estimated that in Germany, between 1400 and 2800 tons of TWP per year are deposited on agricultural land through the application of biosolids.^3^

TWP can introduce a large suite of organic compounds^4,5^ to farmland soils with unknown effects on biota. Aside from rubber and fillers, tires contain additives that are, to date, indispensable for their functionality. These include vulcanization accelerators, activators, plasticizers, processing aids, and antioxidants.^6^ The vulcanization accelerators 1,3-diphenylguanidine (DPG) and benzothiazole (BTZ) have been detected at concentrations in the μg L^–1^ range in rivers.^7^ Although these concentrations are close to the compounds’ predicted no effect concentrations (PNEC), it was shown that at higher concentrations, they are toxic to fish embryos.^8−10^ Similar concentrations have been reported for hexamethoxymethyl melamine (HMMM), a cross-linking agent with over 30 known transformation products.^11,12^ TWP have been implicated as the predominant source of these compounds in rivers.^7^ Not only the parent compounds but also their transformation products, many of which are still unknown, may exert harmful effects on biota. For example, the anti-ozonant *N*-phenyl-*N*′-(1,3-dimethylbutyl)-*p*-phenylenediamine (6PPD) is transformed into a significantly more toxic quinone transformation product (6PPD-q), which is responsible for mortalities of coho salmon through the introduction of street runoff into surface waters, where it has been detected in the concentration range of μg L^–1^.^13,14^

When TWP enter farmland soils, they are expected to release these compounds in the upper soil layers, rather than transporting them to deeper soil horizons.^15^ Once released in the root zone, the compounds may be readily available for root uptake by edible plants, as has previously been shown for various pharmaceuticals that were taken up from reclaimed wastewater and detected at concentrations of up to 300 ng g^–1^ in leafy greens^16^ and plastic-derived phthalates that were detected at maximum concentrations of up to 2.4 μg g^–1^ in cultivation experiments spiked at 500 μg kg^–1^.^17^ It was shown that through the uptake of well water, up to 80 ng g^–1^ of benzotriazole was present in lettuce plants from an agricultural field in California,^18^ but uptake of the broad range of TWP-derived compounds by edible plants has not previously been investigated. Hydrophobic neutral compounds are readily taken up by plant roots, while anionic compounds are mainly repelled by negative charges at cell membranes in the roots.^19,20^ Compounds then migrate through the root tissue toward the xylem either via the symplastic pathway (through the cytoplasm via plasmodesmata) or via the apoplastic pathway (through the intercellular space, i.e., cell walls). Along their transport pathways, hydrophobic neutral compounds may partition into root lipids, thereby hindering their translocation to the leaves of plants. For neutral compounds, the mobility within the plant depends on the compound’s octanol–water partitioning constant (*K*_ow_), with maximum translocation occurring at a log *K*_ow_ of 1.78.^20,21^ For ionic and ionizable compounds, electrostatic interactions with root tissues can also hinder mobility, and *K*_ow_ alone is an insufficient predictor for their mobility. Weak acids and bases can be ionized in the various root cellular compartments, which have different pH values.^22^ Cationic compounds can be retained in the roots due to interactions with negatively charged cell walls, while weak acidic compounds can be subject to ion trapping, in which they diffuse into the cell in their neutral form, and are then trapped as anions in the more alkaline cellular cytoplasm.^22,23^ Compounds that are mobile within the plant tissue will passively move upward with the transpiration stream. However, the transport of organic compounds can exceed transpiration rates due to active transport by transporter proteins, as was shown, e.g., for isothiazolinone, benzotriazole, and mercaptobenzothiazole in *Arabidopsis* plants.^24−26^ Finally, physical properties of the compounds can also affect their mobility. The diffusion of compounds >400 gmol^–1^ through the plant roots is mostly hindered at the Casparian Strip (the suberized center of the root endodermis), preventing them from entering the xylem and hindering them from being translocated into the plant leaves.^20^

The effective accumulation of TWP-derived organic compounds in plants also depends on the plant’s metabolism. Within the plant roots and leaves, plant metabolism generally starts with phase I metabolism: an activation of the compound through hydroxylation, hydrolysis, or dealkylation.^27^ These activation processes can be catalyzed, e.g., by the enzyme cytochrome P450 oxidase mediating the oxidation/hydroxylation of contaminants.^28^ These intermediate transformation products are often short-lived and further transformed by phase II metabolism, in which the activated compound forms a covalent bond with sugars or amino acids (transglycosylation or transamination processes). For compounds already containing −OH or −NH_2_ functional groups, phase I activation may not be necessary because the compound can be directly conjugated at these sites.^20^ Conjugates such as glycosylated metabolites are typically more water soluble than the original compound and can thus be transferred back into the roots and excreted into the external substrate, where subsequent deconjugation can occur.^25,29^ Furthermore, the conjugated compounds can be deposited in plant vacuoles as a detoxification mechanism.^17,26^ The complete metabolization of organic compounds can occur within a few hours.^28^

This study aimed to investigate whether lettuce plants take up TWP-derived compounds, thereby posing a potential risk to human health. We identified transformation products in the plant leaves to understand their fate and possible human exposure to these compounds. Assuming that TWP may be a long-lasting source of TWP-derived compounds to plants, we further investigated the effect of continuous leaching from TWP on the concentration of TWP-derived compounds and their transformation products in lettuce leaves. We show that the monitoring of parent compounds may lead to an underestimation of human exposure concentrations and emphasize the presence of metabolized TWP-derived compounds with largely unknown toxicities in edible plants.

## 2 Methods

### 2.1 Chemicals and Materials

TWP-derived compounds and selected chemical properties are shown in Table S1. DPG (Sigma-Aldrich 43,029), BTZ (Sigma-Aldrich 61,427), 6PPD (Sigma-Aldrich CDS013697), HMMM (TCI Europe T2059), and 6PPD-q (HPC Standards GmbH 687,855) were purchased, and stock solutions were prepared and stored in LC–MS grade acetonitrile at 1 gL^–1^. Ultrapure water was produced by a deionization system (PURELAB Ultra, ELGA LabWater Global Operations, 18.2 MΩ cm). LC–MS grade acetonitrile was purchased from VWR, formic acid from Sigma-Aldrich, and sodium chloride (NaCl) from Merck. Recycled tire granulate with a size distribution of 0.4–0.7 mm was provided by an Austrian tire recycling company that produces tire granulate from used truck and car tires.

### 2.2 Exposure Experiments

Lettuce seedlings of the type *Valerianella locustaL.* were bought from a local garden supply market, removed from the soil, and thoroughly rinsed with tap water. All plants had well-developed roots and 1.6 ± 0.5 g total fresh weight. Each rosette was fixated in a glass vial containing 30 mL of hydroponic nutrient solution prepared with Blusana fertilizer and placed in a growth chamber. The light-period was 16 h day^–1^, the light intensity was 425 μmol m^–2^ s^–1^ (PAR spectrum), and it was 18 °C during the day and 15 °C during the night at 75% relative humidity. After 2 days of equilibration with the nutrient solution, the vials were spiked with HMMM, DPG, and BTZ to a concentration of 1000 μg L^–1^ in the nutrient solution. This concentration was chosen after evaluating three different dosing levels in pre-experiments and was selected to obtain reliable data well above the level of quantification. Due to the degradation in the stock solution, initial concentrations of 6PPD and 6PPD-q were 400 and 200 μg L^–1^, respectively. In a second experimental set, 3 g of tire granulate were added to each vial and placed on a magnetic stirring plate at 350 rpm to keep the particles in suspension. Control samples with plants but without compounds/TWP and control samples with compounds/TWP but without lettuce plants were set up, and all experiments were run in triplicates. All vials were wrapped in aluminum foil to avoid photodegradation of spiked or leached compounds. The nutrient solution in each vial was replenished every day. Plants were harvested after 0.125, 0.25, 0.5, 1, 2, 4, 7, 10, and 14 days, and at each time point, the plants and vials were sacrificed. The roots were rinsed with 2 mL of acetonitrile, which was combined with the rest of the nutrient solution of the respective vial. The roots were dried with lint-free tissue and separated from the leaves, and the fresh weight of roots and leaves were recorded. Roots and leaves were immediately frozen at −20 °C, and after the last harvest, all samples were freeze-dried in one batch.

### 2.3 Extraction of TWP-Derived Compounds

After freeze-drying, the plants’ dry weight was recorded and root and leaf samples were transferred to centrifuge vials. The plant tissues were extracted using a bead beater (Bead Genie, Scientific Industries) with stainless steel beads and 2 mL of acetonitrile for 3 min at 4500 rpm. The samples were centrifuged for 5 min at 5000*g*, and the supernatant was recovered. This extraction was repeated twice with a cumulative volume of 5.5 mL, which was filtered (0.22 μm nylon syringe filters, VWR 514-0066) into clean glass vials for further analysis. Extraction efficiencies from plant material including loss of analytes to the nylon filters were tested with spiked concentrations of 20 and 100 μg L^–1^ and ranged between 87.6 and 103% (mean 95.3 ± 4.7%, Table S2). TWP-derived compounds remaining in the nutrient solution were extracted by liquid–liquid extraction, adding 2 mL of acetonitrile to 3 mL of nutrient solution, adding 300 mg of NaCl for phase separation, and recovering the supernatant. This was repeated once, and the supernatants were combined and filtered (0.22 μm nylon syringe filters) into clean glass vials for analysis. Extraction efficiencies from nutrient solution including loss of analytes to the nylon filters were tested with spiked concentrations of 50 and 500 μg L^–1^ and ranged between 87.2 and 104% (mean 93.8 ± 6.0%, Table S2).

### 2.4 Liquid Chromatography–Mass Spectrometry Measurements

TWP-derived compounds were quantified using ultra performance liquid chromatography coupled to triple quadrupole mass spectrometry (Agilent 6470, hereafter: triple quadrupole-MS) using external standards purchased from Sigma-Aldrich, TCI Europe, and IPC Standards GmbH. Results of the extraction tests imply that the sample matrix did not significantly impact measurements. The limit of quantification (LOQ) was determined as mean concentration of 5 blanks plus 10 times the standard deviation.

To evaluate the uptake and accumulation of the compounds in lettuce roots, the root concentration factor is expressed as
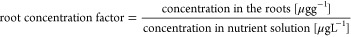
As interactions of the compounds with the root tissue can affect their transport into plant leaves, the translocation factor was calculated as
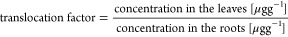
Leaf extracts were additionally measured with an untargeted work flow using ultra performance Orbitrap high resolution mass spectrometry (Thermo Scientific Q Exactive, hereafter: Orbitrap-HRMS) for transformation product analysis. Measurement parameters for both triple quadrupole-MS and Orbitrap-HRMS measurements are reported in the Supporting Information (Sections S1 and S2). To identify transformation products, we first used Compound Discoverer software (Thermo Scientific, Version 3.1.1.12)^30^ (Section S3, Figure S1, Table S5) to filter for compounds with an intensity ratio of at least 5 between the spiked lettuce samples and the control lettuce samples. For BTZ, due to contamination of the control samples, this filter was turned off. The transformation products were expected to be structurally similar to their parent compounds and therefore produce common fragments. Therefore, we used the Molecular Networks tool of Compound Discoverer software to group unknown compounds with MS1 and MS2 spectral similarity (at least two matched centroids and 50% spectral similarity, FISh scoring) to the metabolized TWP-derived compounds.

We additionally used the Generate Expected Compounds tool of Compound Discoverer software to generate a list of possible transformation products of the TWP-derived compounds, based on plausible activation reactions for the TWP-derived compounds^11,31^ and conjugation reactions previously reported in plants (Table S6).^12,26^ We then retroactively screened our data for these precursor masses (within 5 ppm) with the Find Expected Compounds tool. With this approach, we were able to identify additional compounds of interest, which were not present at high enough abundance to trigger MS2 scans in the Orbitrap-HRMS and were thus not identified with our molecular networking approach. For these compounds, we generated MS2 spectra by remeasuring samples using an inclusion list. We manually compared these MS2 spectra to the MS2 spectra of the associated parent compounds, and in the case of at least two fragment matches, we report the compound as a potential transformation product of the original TWP-derived compounds (similar to what the molecular networking tool does automatically).

### 2.5 Sorption to Lettuce Roots

To evaluate the sorption of the TWP-derived compounds by lettuce root cell walls of the epidermis as well as the apoplast, batch experiments were performed. Clean lettuce roots were freeze-dried, ground to fine powder, suspended in nutrient solution (3.4 gL^–1^), and equilibrated overnight. Each compound was spiked to a concentration of 50 and 500 μg L^–1^ in the nutrient solution. The samples were agitated on a reciprocal platform shaker at 125 rpm at room temperature under exclusion of light, followed by centrifugation at 5000*g* and filtration of the supernatant (0.22 μm nylon syringe filter).^20^ The amount of analyte remaining in the nutrient solution after sorption by the lettuce roots was quantified by triple quadrupole-MS analysis to calculate distribution coefficients (*K*_D_) between roots and nutrient solution. The amount of analyte sorbed was normalized to dry root biomass to obtain contaminant concentration in μg g^–1^ of root biomass.

### 2.6 Statistical Analysis

All experiments were run in triplicates, and technical duplicates were analyzed for each sample. All calculations and statistical analyses were done in R, version 4.2.0. To evaluate statistically significant differences in concentrations, ANOVA testing was used. For multivariate comparisons, Tukey’s HSD post-hoc testing was performed and results were visualized with compact letter display (Tables S7–S11).

## 3 Results and Discussion

### 3.1 TWP-Derived Compounds Are Taken Up by Lettuce Roots and Are Translocated into Their Leaves

**Figure 1:**
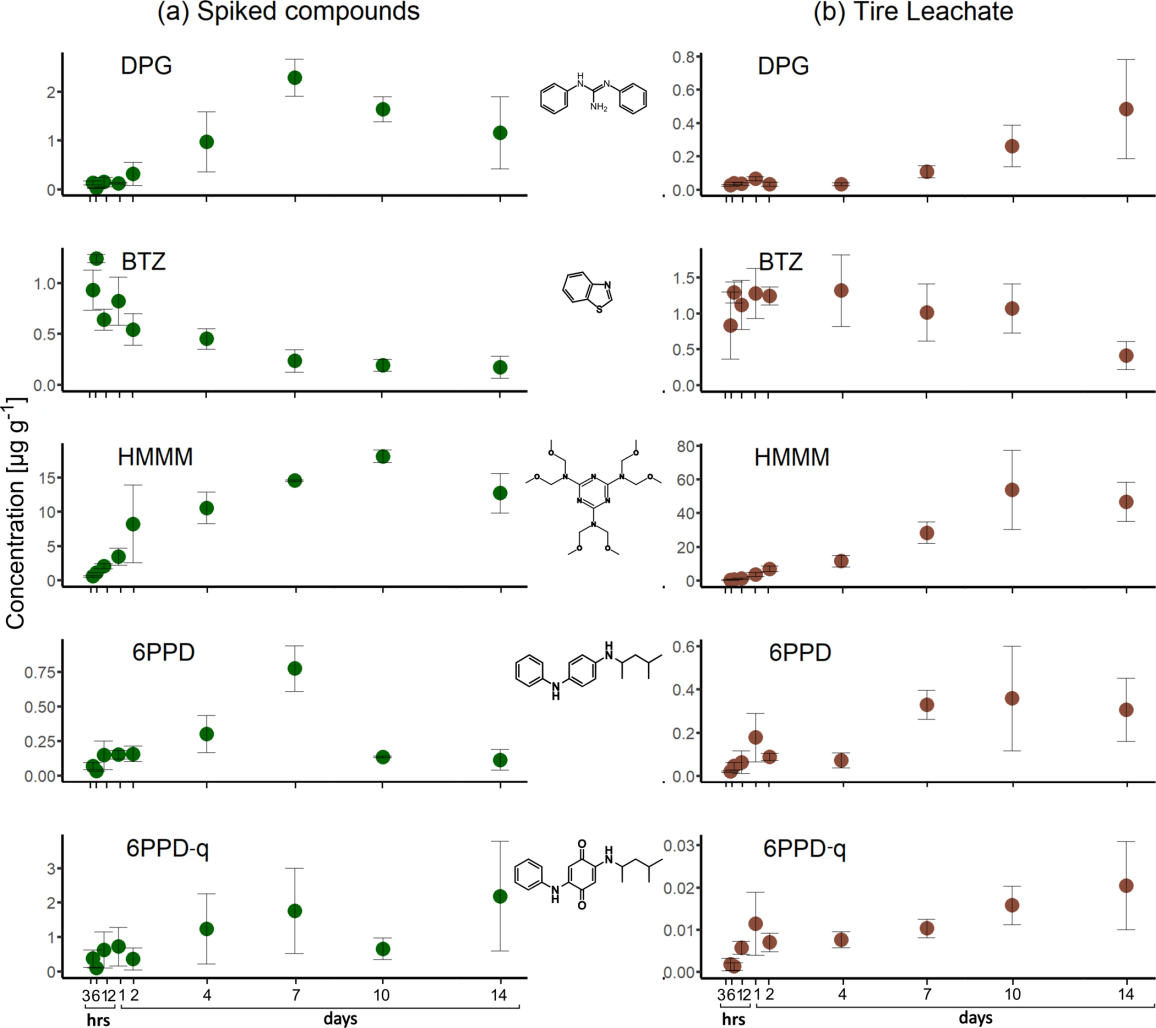
Concentration of TWP-derived compounds per unit biomass in lettuce leaves over 14 days after exposure to (a) a single initial spike (green dots) and (b) continuously replenished leaching from TWP in the nutrient solution (brown dots). Error bars represent the standard deviation from triplicate measurements. All five TWP-derived compounds were taken up into lettuce leaves.

The lettuce plants that were exposed to spiked TWP-derived compounds did not show any signs of phytotoxicity as indicated by the recorded biomass and the absence of visible cues of growth inhibition (Table S12). Moreover, we observed increased depletion (*p* < 0.05) of all TWP-derived compounds from the nutrient solution in the samples containing plants as compared to the control samples (Figure S2, Table S11). DPG, HMMM, and BTZ were stable in the nutrient solution controls over the experimental time span, while the loss of 6PPD and 6PPD-q in the control samples does not account for their depletion when plants were present (Figure S2). Accordingly, all five compounds were detected in roots and leaves of the lettuce plants (Figures 1a and 3).

The concentrations of DPG, HMMM, and 6PPD in lettuce leaves peaked after 7 to 10 days at 2.29, 18.0, and 0.78 μg g^–1^, followed by a subsequent decline to 1.16, 12.7, and 0.11 μg g^–1^, respectively. In contrast, the main uptake of BTZ occurred within the first 6 h (up to 1.24 μg g^–1^), followed by a rapid decrease in its concentration to 0.17 μg g^–1^ thereafter. Concentration decreases were observed for DPG, BTZ, and 6PPD (*p* < 0.05). After 10 days, we observed a decreasing trend in HMMM concentration, which might become significant with longer experimental times (>14 days). Only 6PPD-q showed a continuous increase in concentration over the full 2 weeks of the experiment, with values up to 2.19 μg g^–1^ (Figure 1, Table S8).

Despite different uptake rates, all five parent compounds could be identified in the lettuce leaves, which can be attributed to their low molecular weight. The diffusion of the studied compounds into the xylem and subsequent translocation into leaves was not hindered at the Casparian strip, as the molecular weights of all studied compounds were below the approximate exclusion threshold of 400 gmol^–1^ (Table S1).^20^ Further, experimental *K*_D_ values for lettuce roots were low for all compounds (0.05–0.7 L g^–1^), (Table S13) indicating that the translocation was not entirely hindered by sorption of the compounds to the root tissue and that these TWP-derived compounds can passively transit the plasma membranes without the need of secondary active transport mechanisms. Active transport was found to not be a significant contributor to translocation of cyetpyrafen.^32^ On the other hand, uptake of benzotriazole and 2-mercaptobenzothiazole into *Arabidopsis* plants was shown to exceed the transpiration stream by far.^25,26^ Thus, the fast uptake of BTZ in lettuce plants (Figure 1) may be partly attributed to the contribution of active transporter proteins.

**Figure 2:**
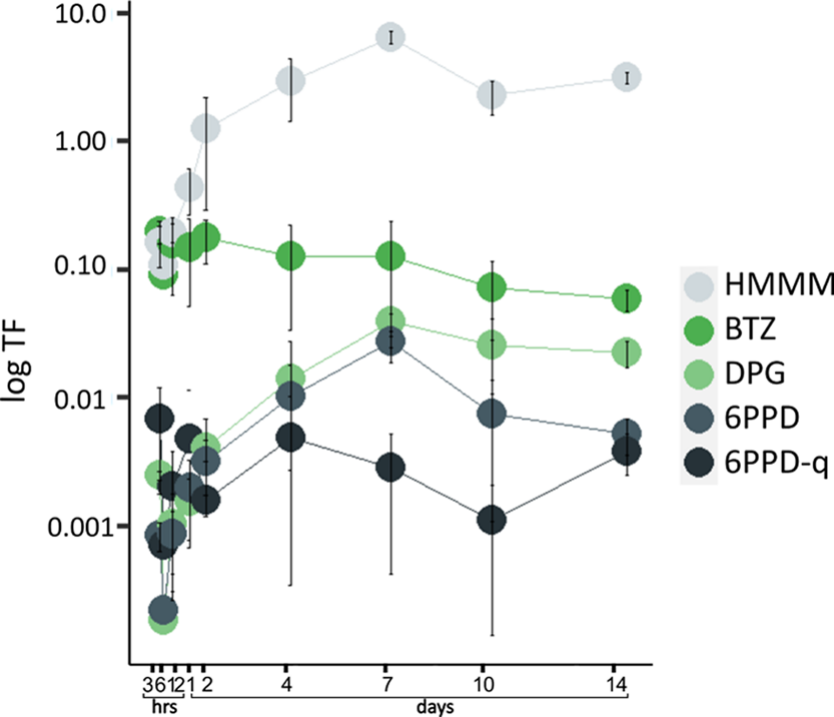
Translocation factors (log TF) of TWP-derived compounds from lettuce roots to the leaves after a single initial compound spike. Error bars represent the standard deviation from triplicate measurements.

However, the peak concentrations and the rate of translocation from roots into leaves differed among the compounds (Figure 1a). At the peak concentration, which was reached after 7–10 days, HMMM had accumulated in the leaves at significantly (*p* < 0.05) higher concentrations than all other compounds, i.e., at a level of ∼20 μg g^–1^, and showed a higher translocation (*p* < 0.05) from roots to leaves (translocation factor > 1) (Figure 2, Table S9). During the first 24 h of the experiment, BTZ showed a similarly high translocation factor, and accordingly, HMMM and BTZ were translocated the fastest from lettuce roots to leaves, showing sharp concentration increases in the leaves within the first day of the experiment. In comparison, the translocation factors of DPG, 6PPD, and 6PPD-q were approximately 2–3 orders of magnitude lower than that of HMMM and the increase in leaf concentration occurred after a slight delay (Figure 1a).

The observed differences in uptake rate and translocation can be explained by differential retention of the compounds in the root tissue. Although the measured *K*_D_ values (Table S13) were low, there were slight differences in affinities of the compounds to the roots. The measured *K*_D_ values followed the order of 6PPD > DPG > HMMM ≅ BTZ, which corresponds to the observed order of translocation factors of the compounds (Figure 2). The lower translocation factors and higher *K*_D_ values for DPG, 6PPD, and 6PPD-q not only explain the lower concentrations in the leaves compared to HMMM but also correspond to their delayed increase in leaf concentration compared to the accumulation of HMMM and BTZ in lettuce leaves within the first day.

**Figure 3:**
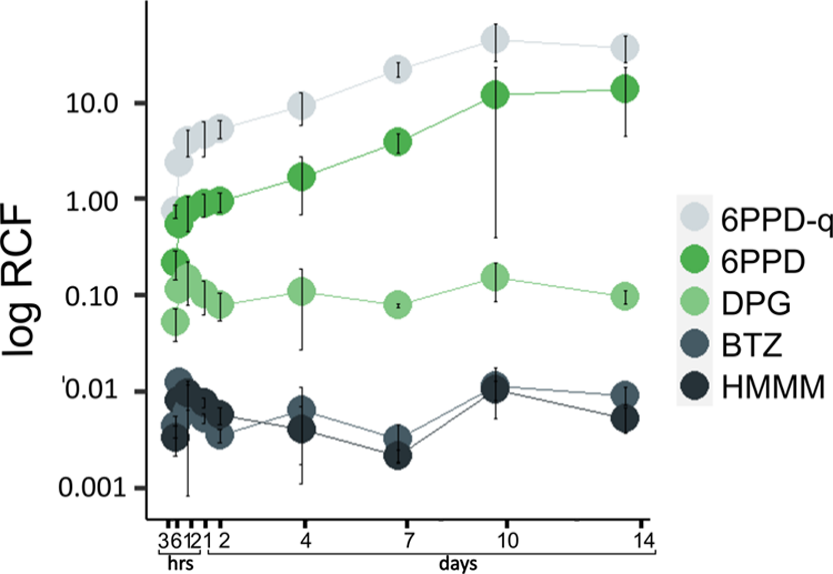
Root concentration factors (RCF) between nutrient solution and lettuce roots of the TWP-derived compounds 6PPD-q (light gray dots), 6PPD (green dots), DPG (light green dots), BTZ (dark gray dots), and HMMM (black dots) after a single initial compound spike. Error bars represent the standard deviation from triplicate measurements.

The *K*_D_ and inversely associated mobilities of TWP-derived compounds in lettuce plants depend on the hydrophobicity and charge of the compounds (Table S1).^23^ Highly hydrophilic compounds barely pass through lipid membranes in the roots and would require transporter (carrier) proteins in the root membranes. On the other hand, highly hydrophobic compounds can be retained by partitioning into root lipids.^21^ The maximum mobility of TWP-derived compounds into and through the roots is therefore expected for neutral compounds with a log *K*_ow_ of 1.78,^21^ which corresponds to that of BTZ (log *K*_ow_ = 2) and HMMM (log *K*_ow_ = 1.6) and explains their high translocation factors. Accordingly, BTZ and HMMM barely accumulated in the roots, resulting in low root concentration factors during the entire experiment (Figure 3). In comparison, the *K*_D_ and therefore the root concentration factor of DPG are approximately one order of magnitude higher. The higher log *K*_ow_ of DPG (2.9) indicates that the retention of DPG in the roots is caused by partitioning into root lipids.^19^ Furthermore, DPG (pK_a_ = 10) is present mostly in its cationic form at the physiological pH of both the apoplast (pH 4–6.3) and the cytoplasm (pH 7.3–8) and can therefore be retained by negative charges of the cell walls in the root apoplast.^22^ The interaction of polyphenols in root tissues with primary amines may explain the higher root retention of DPG compared to HMMM and BTZ.^20^ Although BTZ and HMMM with pK_a_ values of 7.80 and 7.01, respectively, could be similarly cationic in the apoplast, their pK_a_ values are closer to the pH values in the cytoplasmic plant compartments, causing them likely not to be fully ionized or to be neutral in this pH range. Due to their mobility in plant tissue, BTZ and HMMM may passively diffuse through the cell membrane, where the more alkaline pH of the cytoplasm is close to the pK_a_ of both compounds. Thus, BTZ and HMMM in the cytoplasm may be present to a large extent in their neutral form and pass unhindered through the symplastic pathway.

6PPD and 6PPD-q consistently exhibited the lowest translocation factors of all compounds, although the differences in translocation factors between the different compounds were generally small (Figure 2, Table S9). Due to the fast abiotic degradation of 6PPD-q in aqueous solution during the sorption experiments, *K*_D_ values could not be reliably determined. Nevertheless, the root concentration factors of 6PPD and 6PPD-q are several orders of magnitude higher than those of the other TWP-derived compounds, indicating stronger sorption of these compounds to root cell walls (Figure 3). This higher sorption can be explained by their high log *K*_ow_ of 5.6, suggesting strong partitioning into root lipids. In addition, 6PPD-q may be able to form H-bonds between its quinone and root polyphenols.^20,33^

We cannot rule out that the concentration increase of 6PPD-q was, in part, due to transformation from 6PPD, but we do not expect this to be a very relevant transformation in our experiments. The majority of 6PPD-q is formed via ozonation of 6PPD at tire surfaces,^34^ which is unlikely to occur in the plants or nutrient solution. Rather, the continuous increase in foliar concentration of 6PPD-q can be explained by the high root concentration factor retaining 6PPD-q and delaying its translocation to the leaves.

Although plant uptake is strongly compound dependent, the data demonstrate that TWP-derived compounds with a variety of structural differences have the potential to be taken up and translocated to edible plant parts. Despite being partially retained in the roots, even hydrophobic and ionic compounds were translocated to lettuce leaves.

### 3.2 Lettuce Plants Metabolize TWP-Derived Compounds and Accumulate Their Transformation Products in the Leaves

The concentration increases of 6PPD, DPG, BTZ, and HMMM in the lettuce leaves were followed by rapid concentration decreases (Figure 1a). Although the upward flow of the xylem is usually higher than the downward flow of the phloem toward the roots, the analytes may have been partly transported back into the roots.^35^ To confirm that this decrease in concentration of TWP-derived primary compounds in lettuce was not due to the retranslocation of the compounds to the roots or due to depuration (excretion) into the nutrient solution, we calculated mass balances for all the TWP-derived compounds (Figure S3). We found a net mass loss of these compounds in the plant samples compared to the controls, and the decreasing concentration in the leaves suggests that this mass loss can be attributed, at least partly, to metabolism in the leaves. Metabolites may then undergo various fate processes, such as storage, depuration, or further transformation.

6PPD and DPG reached maximum concentrations at one week of exposure, after which 6PPD declined to concentrations close to the LOQ, while DPG was metabolized to a lesser extent (Figure 1a). DPG and 6PPD both contain secondary amines, a functional group that is generally prone to undergo biotransformation reactions^36^ and has been shown to be sites of, e.g., sugar conjugation (transglycosylation) in plants.^26^ Thus, DPG and 6PPD may be directly conjugated (i.e., without the need for an activation step),^26,37^ which could explain the relatively rapid degradation of DPG and 6PPD in lettuce leaves. HMMM reached a maximum concentration after ∼10 days and then decreased, though much more slowly than the other TWP-derived compounds. The slower degradation rate, along with the fact that HMMM has no functional groups directly amenable to conjugation reactions, suggests that HMMM first undergoes a phase I activation reaction, which can be rate-limiting rather than undergoing direct conjugation.^38^ 6PPD-q did not appear to reach a maximum concentration within the course of the experiment. BTZ displayed the fastest metabolism, reaching its maximum foliar concentration after 6 h and then decreasing to concentrations close to the LOQ for the rest of the experimental duration.

To gain an insight into the fate of the TWP-derived compounds in plants, we measured the leaf extracts with Orbitrap-HRMS, following an untargeted approach. With this approach, we detected 19 potential transformation products of the original five TWP-derived compounds, identified to varying degrees of confidence.^39^ All of these compounds were present at elevated signals in the spiked plant extracts compared to the controls and were structurally related to the original TWP-derived compounds (Section S4, Table S14).

6PPD and DPG were readily conjugated. We tentatively identified one of the transformation products of DPG as an acetate conjugate (TP_DPG_270) and the only detected transformation product of 6PPD as a 6PPD-glucose conjugate (TP_6PPD_431).

**Figure 4:**
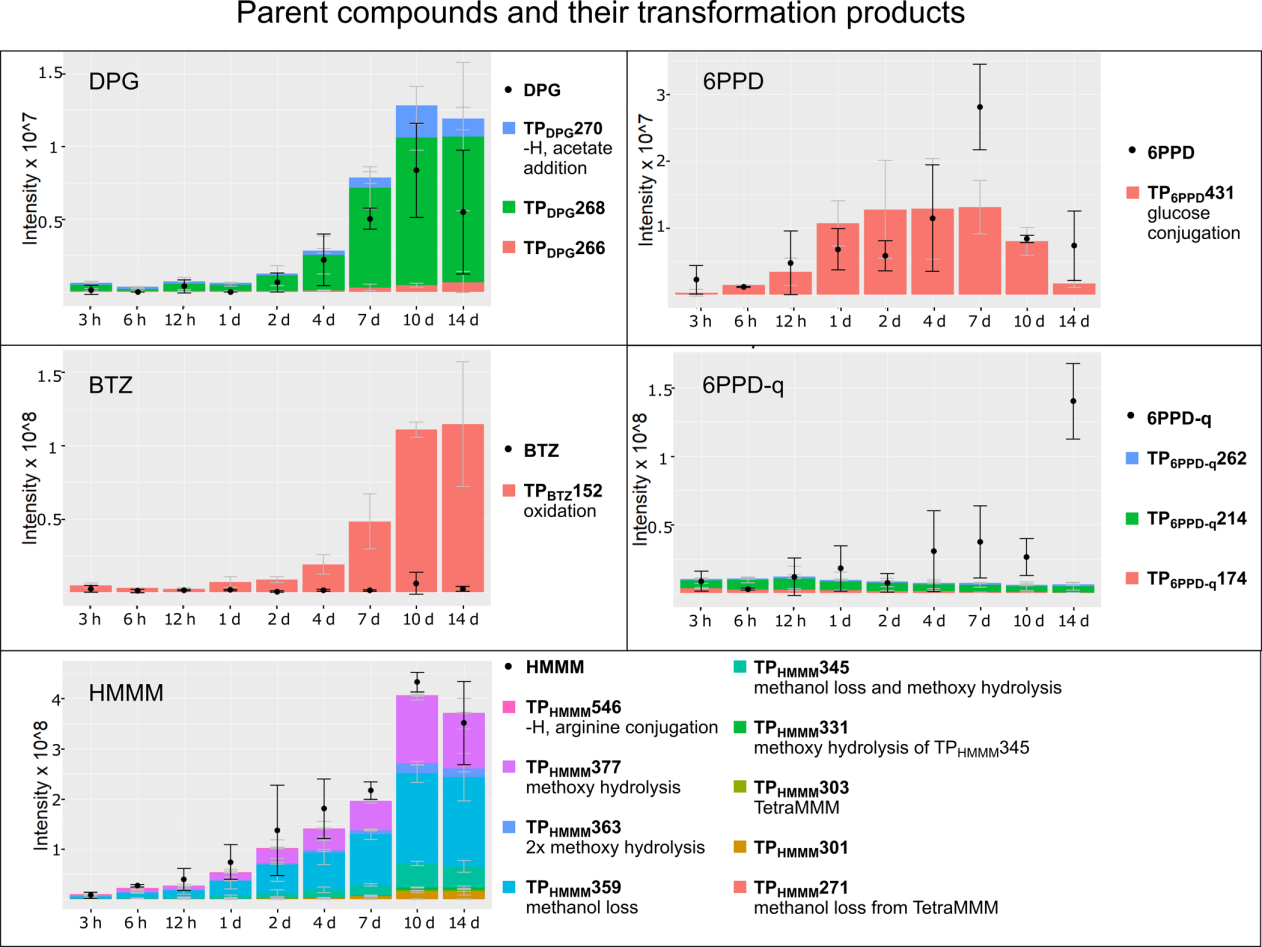
Ion intensities of TWP-derived compounds and their transformation products over time as measured by Orbitrap-HRMS. Black dots represent the measured intensity of the parent compounds, while the stacked bar plots represent the cumulative intensities of the transformation products.

DPG was more stable in a conjugated form, as the measured intensities of its conjugates increased sharply at around 7 days and did not decrease substantially thereafter (Figure 4, Table S10). 6PPD-glucoside, on the other hand, increased and subsequently decreased (*p* < 0.05) in concentration, suggesting further metabolism of the 6PPD-glucoside or alternatively excretion of the conjugated compound. Excretion of glucoside conjugates has been previously demonstrated.^40,41^ Conjugation of phytotoxic compounds creates a more water-soluble molecule, which plants can either excrete or store in their vacuoles, or which accumulate through sorption to cell walls, thereby immobilizing the compound and preventing toxic effects to the plant.^31,37^ Once in the vacuole, conjugated xenobiotics have been shown to undergo further degradation reactions.^31,42^ In this study, the nutrient solution controls showed that DPG was highly stable in water, while 6PPD degraded very quickly (Figure S2). This suggests that the degradation of conjugated compounds in plants may be related to the overall stability of the unconjugated compounds. Since we did not monitor the hydroponic solution for transformation products, we cannot differentiate between excretion and/or further transformation of 6PPD-glucoside. On the other hand, DPG conjugate stability means that DPG could still be present (albeit in its conjugated form) in edible plants at the time of human consumption. This study contributes to the growing body of work demonstrating that monitoring of parent compounds only may lead to an underestimation of exposure risk by ignoring the presence of metabolites, a phenomenon known as “metabolite masking”.^26,40,43^

6PPD-q is inherently unstable, as we measured rapid degradation in the nutrient solution controls. Interestingly, 6PPD-q appeared to be more stable in the plants, displaying a continuously increasing concentration in the leaves for the entire 2 weeks of the experiment (Figure 4). We identified three potential transformation products of 6PPD-q (TP_6PPD-q_214 with monoisotopic mass 213.20858, TP_6PPD-q_174 with monoisotopic mass 173.12001, and TP_6PPD-q_262 with monoisotopic mass 261.13555). To the best of our knowledge, these masses have not previously been reported as transformation products for 6PPD or 6PPD-q. All three transformation products were stable within the plants, with only TP_6PPD-q_174 displaying a slight decrease (*p* < 0.05) in measured intensity over time. The increased stability of 6PPD-q metabolites compared to the 6PPD metabolite may be related to the distinct transformation processes. While glycosylation was a major fate process for 6PPD, leading to potential excretion of the conjugated compound, conjugation was not a major fate process for 6PPD-q.

HMMM was also metabolized by lettuce plants, and we could identify 11 potential transformation products of HMMM. After reaching the maximum concentration at 10 days, the transformation products of HMMM did not decrease significantly (Figure 4, Table S10). In general, HMMM undergoes sequential degradations via only three reaction steps (hydrolysis of a methoxy group, formaldehyde elimination, and *N*-methylol oxidation to aldehyde).^11,12,44^ Although there is growing evidence that the *N*-methylol oxidation to aldehyde reaction is biologically catalyzed,^11,12^ we did not observe any transformation products formed via this reaction in the lettuce plants. Rather, the hydrolysis of a methoxy group was the most relevant reaction, producing two of the four major transformation products (TP_HMMM_377 and TP_HMMM_363), both of which have been ubiquitously detected in urban rivers.^11,45^ Surprisingly, the transformation product measured at the highest abundance in the lettuce leaves (TP_HMMM_359) was previously unreported, with an exact monoisotopic mass of 358.19526 and a proposed molecular formula of C_14_H_26_N_6_O_5_. A molecule with this molecular formula cannot be formed via any of the known HMMM transformation reactions, including those that have been shown to be catalyzed by bacteria and fungi.^11,12^ This may be due to a reaction pathway catalyzed within plants. Based on the proposed molecular formula of this transformation product, we reason that the reaction might release a methanol from HMMM. We observed the same neutral loss between known transformation product TetraMMM and TP_HMMM_271, implying that TP_HMMM_271 was formed via the same methanol loss pathway from TetraMMM. The transformation product with the third highest intensity is also previously unreported (TP_HMMM_345) and may be a secondary transformation product of TP_HMMM_359 via the known methoxy hydrolysis reaction. Although we identified further transformation products, including a probable arginine conjugate of HMMM (TP_HMMM_546), they were measured at much lower intensities than the aforementioned transformation products. Although measured at lower intensity than other transformation products, the tentative identification of an arginine conjugate of HMMM is worth noting. Amino acid conjugates are not excreted as easily as sugar conjugates due to the locations of the respective enzymatic reactions,^41,46^ which may imply long-term stability of TP_HMMM_546. This paper joins a growing body of evidence showing that amino acid conjugation is a relevant biological transformation for xenobiotics.^12,24,25,40^ Some of these novel transformation products of HMMM may be uniquely formed within plants, and we measured four transformation products of HMMM with high intensity, all of which were stable in the leaves over the duration of our experiment.

BTZ was metabolized remarkably quickly in the lettuce leaves. We identified one transformation product of BTZ, TP_BTZ_152 (exact monoisotopic mass of 151.00881, unequivocal molecular formula: C_7_H_5_NOS), which was measured at very high intensity. The measured intensity of TP_BTZ_152 increased continuously over the course of the experiments, even after BTZ had been depleted (Figure 4). The fact that BTZ was stable in nutrient solution (Figure S2) but transformed so rapidly to TP_BTZ_152 in the leaves suggests that the reaction was catalyzed by a highly active catabolic enzyme present in the plants. 2-Mercaptobenzothiazole has also been shown to be transformed very rapidly in plants.^26^ Because of its structural similarity to purines, BTZ may have entered the purine catabolic pathway, as has been previously shown for benzotriazole in the tryptophan pathway.^25^ The exact mass difference between TP_BTZ_152 and BTZ implies the addition of an oxygen atom, which could have been catalyzed by xanthine dehydrogenase, an enzyme that oxidizes the pyrimidine ring of hypoxanthine to xanthine. This reaction would also be feasible for BTZ and would produce the observed compound TP_BTZ_152 (Table S14). Purines are catabolized as an energy source in plants,^47^ and xanthine dehydrogenase is highly active in the leaves of many plant species.^48^ Therefore, the presence and gene expression of this enzyme in plants could explain the rapid transformation of BTZ to TP_BTZ_152 in plant leaves. In the following step of purine catabolism in plants, xanthine dehydrogenase catalyzes the oxidation of the imidazole ring. However, the stability of TP_BTZ_152 and the fact that we did not detect this next oxidation product suggest that BTZ is not further metabolized by the purine catabolic pathway. An alternative hypothesis for the rapid formation of TP_BTZ_152 is oxidation of the reduced sulfur in benzothiazole, as has been shown for benzisothiazolinone.^24^ The metabolism of BTZ that we demonstrate here may have wider implications on the plants’ biochemical processes, as has been recently shown for benzisothiazolinone.^24^ Future work should monitor the activities of relevant enzymes, or the up- or down-regulation of endogenous plant compounds. Regardless of the site of oxidation, TP_BTZ_152 was likely a stable product of BTZ metabolism and is expected to accumulate in lettuce leaves during the growth period.

### 3.3 Leaching from Tire Wear Particles (TWP) Continuously Replenishes Metabolized Compounds in Plants

While some of the transformation products of TWP-derived compounds accumulated in lettuce leaves, the concentration of the parent compounds quickly decreased. However, with TWP as major source of these contaminants, they are likely continuously supplied over the plant’s growth period. Therefore, in a second scenario, we exposed lettuce plants to TWP, which continuously released the five compounds into the nutrient solution (Figure S2). In spite of substantial compound concentrations leaching from the particles, we did not observe phytotoxic effects of TWP or their leachates on lettuce plants, as confirmed by visual inspection of the experimental plants as well as the recorded biomass (Table S12).

Compared to the treatment with a single initial TWP-derived compound spike, the concentrations of all compounds in lettuce leaves were more stable or continuously increased when exposed to TWP due to the resupply from constant leaching of TWP (Figure 1b). The concentration of DPG, which was degraded following a peak in concentration after 7 days in the spike experiment (Figure 1a), continuously increased until the end of the experiment when plants were exposed to TWP (Figure 1b). The concentrations of HMMM and 6PPD remained stable until the end of the experiment instead of decreasing after 7 days, and the concentration of BTZ, which was otherwise metabolized within the first day of the experiment, decreased much later and more slowly when exposing lettuce plants to TWP (Figure 1b).

These TWP-derived compound concentrations in lettuce leaves resulted from compound uptake, translocation, and metabolism and also the differential leaching rates of the compounds from TWP. The leaching rate of DPG remained stable over the first 7 days and then decreases only slightly (Figure S2), and this continuous resupply of DPG into the nutrient solution led to a constant concentration increase in lettuce leaves. In comparison, the leaching rates of HMMM and BTZ were particularly high in the beginning and therefore accumulated in lettuce leaves to much higher concentrations than DPG. However, the leaching rates of HMMM and BTZ plateaued after approximately 4 days, which resulted in a concentration decrease in the leaves as soon as the leaching rate and plant uptake rates declined below those of plant metabolism of those compounds. 6PPD and 6PPD-q instantaneously decompose in the nutrient solution once they leach from TWP, but even so, they showed a more continuous concentration increase in the leaves compared to the spike experiment.

In the soil environment, the leaching behavior of TWP likely differs from the hydroponic experiments, but tire additives are expected to migrate through the TWP and leach out.^49^ For example, amine stabilizers such as 6PPD are designed to freely migrate through the tire rubber toward the surface, allowing them to serve as anti-ozonants,^50^ with the side-effect that they continuously leach into the environment. DPG, BTZ, and HMMM are used as vulcanization accelerators and cross-linking agents, which requires a good dispersibility in rubber,^51^ so that the compounds that are not consumed during vulcanization^52^ leach readily into the environment, making them available for plant uptake.

## 4 Environmental Implications

The uptake and accumulation of five TWP-derived compounds by lettuce plants and the subsequent translocation of the compounds into leaves may be of concern to consumers, particularly because lettuce plants are eaten raw. The fact that even the hydrophobic TWP-derived compounds, 6PPD and 6PPD-q, were readily taken up by lettuce is concerning as it shows that uptake is not restricted to specific molecular characteristics, such as molecular weight and hydrophobicity.

While 6PPD and DPG were likely conjugated by, e.g., transglycosylation, HMMM, 6PPD-q, and BTZ were degraded via various pathways. We here for the first time report conjugation of TWP-derived compounds in edible plants, and we further propose novel transformation products of HMMM and 6PPD-q, including an amino acid conjugate of HMMM. With the exception of 6PPD, plant metabolism formed more stable products that accumulated in lettuce leaves.

Conjugation reactions are generally reversible, and deconjugation has been demonstrated in the human digestive system.^40^ Especially in the case of DPG, conjugation implies that DPG is preserved in the edible part of lettuce plants, and humans could be exposed to it during consumption following deconjugation. For the other TWP-derived compounds with stable transformation products, humans may be exposed to these transformation products, with unknown toxicities. Further work is needed to determine the toxicity of TWP-derived compounds and their transformation products and to determine the environmental levels of these compounds in agricultural products.

The uptake of TWP-derived compounds was studied for exposure conditions in hydroponic cultures. Under environmental conditions, the compounds can be sorbed to soil constituents, decreasing the bioavailability of these compounds in soils.^53^ Other compounds such as pharmaceuticals were shown to sorb to the soil matrix and to quickly equilibrate with soil pore water. This process is highly dependent on soil properties, as well as on compound properties. The charge state of positively ionizable compounds (HMMM, 6PPD, DPG, and BTZ) is determined by soil pH, and in the case that the compounds are present in cationic form, sorption is controlled by the soil’s cation exchange capacity.^54^ The availability of neutral compounds is determined by the soil’s organic carbon content and the compound’s log *K*_ow_.^54^ For example, although 6PPD-q displayed a relatively high root concentration factor under hydroponic conditions (Figure 3), in soils with a high organic carbon content, it is likely that due to the compound’s log *K*_ow_ of 5.0–5.5, 6PPD-q is expected to remain bound to soil rather than being available for plant uptake. Biosolids have a high organic carbon content, which may decrease the availability of neutral tire-derived compounds for plant uptake.^55^ Additionally, co-contaminants could also affect plant uptake. For example, cellular stress induced by mercury was demonstrated to reduce plant uptake of oxytetracycline.^56^ The growth stage of the plant may also impact the accumulation of tire-derived compounds. It has been shown that premature cabbage takes up pharmaceuticals and chemicals derived from personal care products at higher amounts than mature cabbage.^57^ If plants are exposed to tire-derived compounds during early growth stages, uptake may be even higher than what we have demonstrated here, and stable metabolites may accumulate over the plants’ entire life cycle. Under field conditions, further degradation of the compounds may occur over the growth cycle of lamb’s lettuce, which spans over approximately 1 month.^58^ However, the observed stability of the identified transformation products after 2 weeks suggests that even considering longer growth cycles in the field, these transformation products may still be present at harvest.

Organic compounds generally accumulate to a lesser extent in fruit vegetables than in leafy greens or root vegetables^16,57^ because root-shoot transport of organic compounds is assumed to be mostly via xylem, and xylem influx to fruits is limited.^59^ Studies comparing uptake of organic compounds by multiple crops have found that for some compounds, leafy greens such as lettuce and spinach are the strongest accumulators, while other compounds show higher accumulation in root vegetables.^16,57^ In this study, we also demonstrated that preferential accumulation in roots or leaves is compound-dependent. Compounds such as 6PPD-q, which were found to accumulate in lettuce roots, may be of more concern in root vegetables, such as carrots or radish.

The dosing levels used in the experiments were chosen rather high compared to environmentally relevant doses.^18^ A change in the dosing level was previously shown to linearly correlate with the uptake of carbamazepine and diclofenac by lettuce,^60^ and pre-experiments conducted in the course of this study demonstrated similar trends at different dosing levels. On the other hand, the assimilation of 2-mercaptobenzothiazole was higher at lower exposure concentrations^26^ so that a slightly different response at environmentally relevant concentrations may not be excluded. An unresolved, yet critical aspect may be the degradation of these compounds by soil microbial communities before they are taken up by the plants, which should be investigated in the future.

Depending on the input pathway to agricultural fields, some TWP-derived compounds may leach from TWP before they enter the soil. For air-borne TWP, compounds may be lost through volatilization during atmospheric transport, but the majority of the compounds are expected to be released once TWP enter the agricultural soils and come in contact with soil pore water. There, the compounds are released over time and can pose a long-lasting source of toxic TWP-derived compounds to edible plants. In contrast, TWP that are deposited on the road are exposed there to sunlight and rain before entering a (mixed) sewer system and then a WWTP.^7,61^ In this case, TWP may have already released substantial amounts of the compounds, due to prolonged contact with the aqueous phase, and therefore not generate a continuous supply on agricultural fields. However, the compounds may be introduced to agricultural fields if wastewater, which has been shown to carry large amounts of TWP-derived compounds,^7,44^ is used for irrigation. In this study, we used TWP from recycled tires. Despite the previous usage and environmental exposure of this recycled tire material, it leached considerable amounts of compounds. The leaching from aged tire material indicates that intraparticle diffusion of DPG, HMMM, and BTZ, i.e., their migration to the tire surface, can limit the release of these compounds during their transport in the environment. Thus, upon reaching agricultural fields, TWP may continue leaching tire additives, making them accessible for plants and entering the human food web.

This study showed that TWP may be a continuous source of TWP-derived compounds to edible plants and transformation products accumulate in the leaves of lettuce. This may become critical if regulatory thresholds are only defined according to the concentrations of original TWP-derived compounds, while underestimating the sum of parent compounds and transformation products with up to date largely unknown toxicities.

## Supporting Information Available

Additional method specifications (compound information, analytical methods, data analysis, statistical analysis), supporting data (identified transformation products/spectra, sorption data, recoveries, biomass monitoring), and supporting figures (transformation product identification, nutrient solution concentrations, mass balances, HRMS spectra) (PDF)
