# Supplementary material for: Uptake, Metabolism, and Accumulation of Tire Wear Particle-Derived Compounds in Lettuce
Source: Environ Sci Technol. 2022 Dec 28;57(1):168–78. doi: 10.1021/acs.est.2c05660 (PMC9835885; doi:10.1021/acs.est.2c05660)
Supplement: Supplementary file 1 — es2c05660_si_001.pdf [file es2c05660_si_001.pdf]

Supporting Information

Uptake, metabolism and accumulation of tire wear particle-derived compounds in lettuce

Stephanie Castan[a,b†], Anya Sherman[a,b,c†], Ruoting Peng[a,b], Michael T. Zumstein[a], Wolfgang Wanek[d], Thorsten Hüffer[a,c], Thilo Hofmann[a,c*]

a) University of Vienna, Centre for Microbiology and Environmental Systems Science, Environmental Geosciences EDGE, 1090 Vienna, Austria. *thilo.hofmann@univie.ac.at

b) University of Vienna, Doctoral School in Microbiology and Environmental Science, 1090 Vienna, Austria.

c) University of Vienna, Research Platform Plastics in the Environment and Society (PLENTY), 1090 Vienna, Austria

d) University of Vienna, Centre for Microbiology and Environmental Systems Science, Division of Terrestrial Ecosystem Research, 1030 Vienna, Austria.

† These authors contributed equally.

Contents

Table S1. Physicochemical properties of the TWP-derived compounds investigated in this study, including structure, exact mass (g mol^{-1}), dissociation constant (pK_a), and octanol-water partition coefficient ($logK_{ow}$). N/A: not available 6

Table S2. Extraction test recoveries in % for nutrient solution and plant material 7

Section S1. Triple quadrupole-MS analysis 8

Table S3. Method specifications for triple quadrupole-MS measurements 9

Section S2. Orbitrap-HRMS analysis 10

Table S4. Method specifications for Orbitrap-HRMS measurements 11

Section S3. Compound Discoverer analysis 12

Figure S1: Compound Discoverer workflows used for the Molecular Networking approach (top) and Expected Compounds approach (bottom). 14

Table S5. Compound Discoverer parameters 15

Table S6. Transformations for generating expected compounds 16

Table S7. Concentration of TWP-derived compounds per unit biomass in lettuce leaves: Results of Tukey's HSD multiple comparisons across compounds for each time point. Different lowercase letters indicate statistically significant differences between compounds ($p<0.05$) 18

Table S8. Concentration of TWP-derived compounds per unit biomass in lettuce leaves: Results of Tukey's HSD multiple comparisons across time points for each compound.

Different lowercase letters indicate statistically significant differences between time points (p<0.05).........19

Table S9. Translocation factors of TWP-derived compounds from lettuce roots to the leaves after a single initial compound spike: Results of Tukey's HSD multiple comparisons across compounds for each time point. Different lowercase letters indicate statistically significant differences between compounds (p<0.05). NA indicates insufficient data for statistical comparisons. 20

Table S10. Ion intensities of TWP-derived compounds and their transformation products over time: Results of Tukey's HSD multiple comparisons across compounds for each time point. Different lowercase letters indicate statistically significant differences between compounds (p<0.05).........21

Table S11. Concentrations of TWP-derived compounds in the nutrient solution with and without lettuce plants: Results of ANOVA testing for each compound and time point. Asterisk (*) indicates statistically significant differences between samples with and without lettuce plants (p<0.05). 22

Table S12. Biomass of lettuce plants at the beginning and end of the experiment. 23

Figure S2. Concentrations of TWP-derived compounds in the nutrient solution with lettuce plants (● green dots) or without plants (● blue dots) over 14 days after exposure to (a) a single initial TWP-derived compound spike and (b) continuously replenished leaching from TWP in the nutrient solution. Error bars represent the standard deviation from triplicate measurements. 24

Table S13. K_D values of TWP-derived compounds in lettuce root tissue at initial aqueous concentrations of 50 and 500 μgL^{-1}.........25

Figure S3. Mass of DPG, BTZ, HMMM and 6PPD in the nutrient solution (blue bars ■), roots (brown bars ■) and in lettuce leaves (green bars ■) relative to the total mass added at the start. Abiotic controls without plants are represented as black dots (●). 26

Section S4. Transformation products of TWP-derived compounds..27

Table S14. Transformation products of TWP-derived compounds..28

Figure S4: HRMS Spectra parent compounds and transformation products for A)BTZ, B)6PPD, C)6PPD-q, D)HMMM, and E)DPG. 57

Table S1. Physicochemical properties of the TWP-derived compounds investigated in this study, including structure, exact mass (g mol^{-1}), dissociation constant (pK_a), and octanol-water partition coefficient (logK_{ow}). N/A: not available

Compound	DPG	BTZ	HMMM	6PPD	6PPD-quinone
Structure	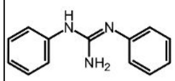	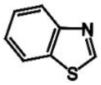	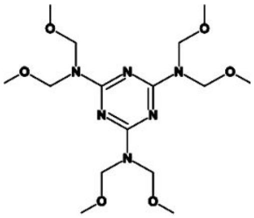	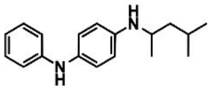	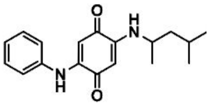
Exact mass (g mol^{-1})	211.11034	135.01403	390.22129	268.19311	298.16702
pK_a	10.12[24]	7.8[25]	7.01[7]	6.7[26]	N/A
logK_{ow}	2.9[24]	2[27]	1.6[8]	5.6[10]	5-5.5[10]

Table S2. Extraction test recoveries in % for nutrient solution and plant material

Matrix	Tested concentration [µg L^{-1}]	DPG	BTZ	HMMM	6PPD	6PPD-q
Nutrient	50	103	87.2	91.6	89.3	104
solution	500	91.8	97.9	87.7	91.1	94.3
Plant	20	96.6	103	92.1	90.8	87.6
material	100	95.4	93.5	95.9	96.1	102

Section S1. Triple quadrupole-MS analysis

The TWP-derived compounds extracted with acetonitrile were analyzed by ultra-performance liquid chromatography - triple quadrupole mass spectrometry (Agilent 1290 Infinity II - Agilent 6470, hereafter: triple quadrupole-MS) using a C18 column (Acquity HSS T3, 1.8 µm, Waters) in the positive ionization mode (capillary voltage: 2500 V) via multiple reaction monitoring (MRM). The LC flow rate was 0.6 mL min^{-1} at a column temperature of 40 °C and the injection volume was 1 µl. The mobile phase consisted of ultrapure water (Phase A) and acetonitrile (Phase B), both containing 0.1% formic acid. The eluent gradient was held at 95% Phase A for one minute, then set that the contribution of Phase A decreased from 95 to 5% over 7 min, was held at 5% for 1 min and increased again to 95% over the last 2 min. Electrospray ionization (ESI) ionization was achieved at gas temp 240°C, gas flow 5 $Lmin^{-1}$. The nebulizer was set to 30 psi; sheath gas to 250°C, 11 $Lmin^{-1}$. Quantification was achieved by external calibration standards prepared in acetonitrile (0.1 to 1500 μgL^{-1}). Transitions from precursor ions to product ions are specified in Table S1.

Table S3. Method specifications for triple quadrupole-MS measurements

Compound name	Precursor ion m/z	Product ion m/z	Fragmentor voltage (V)	Collision energy (V)	Cell accelerator voltage (V)	Retention time (min)
6PPD	269	184	150	45	5	3.34
6PPD	269	107	150	45	5	3.34
6PPD	269	93	150	45	5	3.34
6PPD-quinone	299	256.1	112	23	5	5.14
6PPD-quinone	299	241	112	31	5	5.14
6PPD-quinone	299	215	112	15	5	5.14
6PPD-quinone	299	187	112	31	5	5.14
Benzothiazole	136	109	150	31	5	2.72
Benzothiazole	136	77	150	27	5	2.72
Benzothiazole	136	65	150	38	5	2.72
DPG	212	195	150	20	5	1.7
DPG	212	119	150	20	5	1.7
DPG	212	94	150	20	5	1.7
HMMM	391	283	150	15	5	3.17
HMMM	391	253	150	23	5	3.17
HMMM	391	207	150	19	5	3.17
HMMM	391	177	150	35	5	3.17

Section S2. Orbitrap-HRMS analysis

The leaf extracts were further analyzed by high-performance liquid chromatography – Orbitrap high resolution mass spectrometry (Thermo Scientific Ultimate 3000 – Thermo Scientific Q Exactive^{TM}, hereafter: Orbitrap-HRMS) The liquid chromatography was operated using an LPG-3400SD pump. Phase A was ultrapure water with 0.1% formic acid. Phase B was acetonitrile with 0.1% formic acid. Phase A was held at 95% for one minute, then decreased to 5% over 11 minutes. It was then held at 5% for 2 minutes, before increasing back to 95% over 2 more minutes. 5 minute equilibration time was included between each sample. Autosampling was performed with a WPS-3000 autosampler. The draw speed was 0.5 μLs^{-1}, with a 0.003 s draw delay. Dispense speed was 8.333 μLs^{-1}. The same C18 column (Acquity HSS T3, 1.8 µm, Waters) as from the triple quadrupole-MS was used and maintained at 40 ^{0}C with a TCC-3000 column oven.

The Q Exactive – Orbitrap HRMS (Thermo Scientific, Vienna, Austria) was operated in Full MS / dd-MS^2 mode (exact parameters to be found in Table S2). An initial screening run was performed on six extracts of control plants, and on six extracts of the spiked plants harvested after 14 days. The Compound Discoverer software (Thermo Scientific, Vienna, Austria) was used to annotate compounds with an intensity ratio of less than 5 between the control and spiked plants as background compounds. This list of background compounds was exported and used as an exclusion list for the following measurements. All control leaf extracts and spiked leaf extracts from all time points were measured with the Orbitrap-HRMS and analyzed with Compound Discoverer.

Table S4. Method specifications for Orbitrap-HRMS measurements

**Orbitrap-HRMS Parameters**	
**General**	
Runtime	1.5 to 11 min
Polarity	Positive
Default charge state	1
**Full MS**	
Resolution	70,000
AGC target	$3x10^6$
Maximum IT	100 ms
Scan range	70 to 1000 m/z
**dd-MS^2**	
Resolution	17,500
AGC target	$2x10^4$
Maximum IT	120 ms
Loop count	5
TopN	5
Isolation window	0.4 m/z
(N)CE / stepped nce	20, 30, 40
**dd Settings**	
Minimum AGC target	$1x10^2$
Intensity threshold	$8.3x10^2$
Exclude isotopes	On
Dynamic exclusion	5.0 s
If idle…	Pick others

Section S3. Compound Discoverer analysis

Compound Discoverer 3.1.1.12 was used. Two separate workflows were applied, one for the Molecular Networks analysis, and one for the Expected Compounds analysis. Both workflows, and their associated parameters are presented in Figure S1 and Figure S2, respectively. Molecular networking was used to identify structurally related compounds producing common fragments (see also the main text section 2.4). Transformation products of the parent compounds were then inferred based on the MS2 spectra, and in some cases, by comparison with the list of expected compounds. For example, the shared fragments of DPG and its detected transformation products, i.e., $TP_{DPG}266$ (proposed molecular formula: $C_{16}H_{15}N_3O$), $TP_{DPG}268$ (proposed molecular formula: $C_{15}H_{13}N_3O_2$), and $TP_{DPG}270$ (proposed molecular formula: $C_{15}H_{15}N_3O_2$) support their structural relation to DPG. These transformation products had masses higher than the parent compound, implying conjugation reactions. For $TP_{DPG}268$, the exact monoisotopic mass of DPG was found in the MS2 spectra. This implies that the overall structure contained an intact DPG molecule covalently linked to an endogenous biomolecule, which was then fragmented by Orbitrap-HRMS ion fragmentation, producing DPG as a fragment.[42] For $TP_{DPG}270$, the exact precursor mass matches the predicted mass for acetate conjugation, suggesting acetate as the endogenous ligand. Following the same line of reasoning, we suggest that the transformation product of 6PPD that we measured is a 6PPD-glucose conjugate.

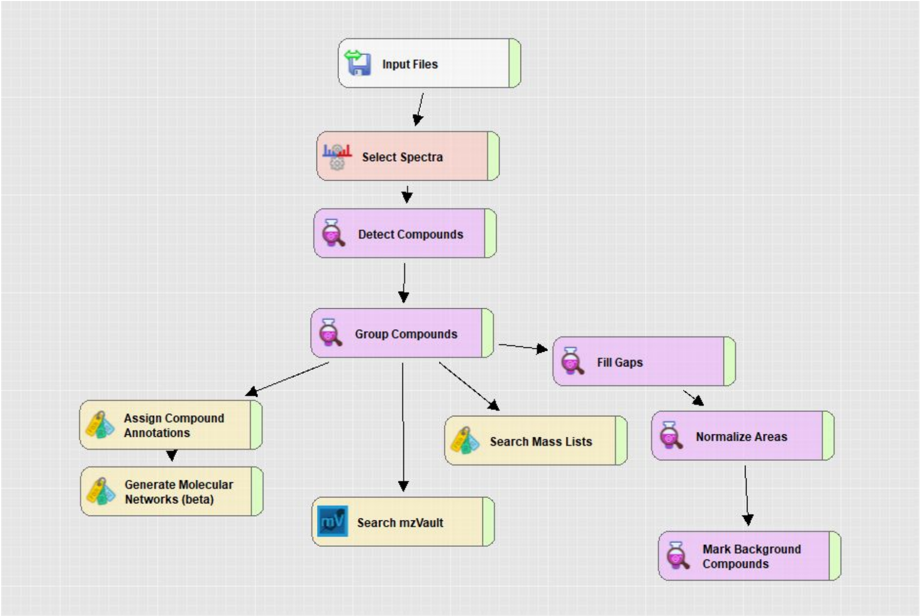

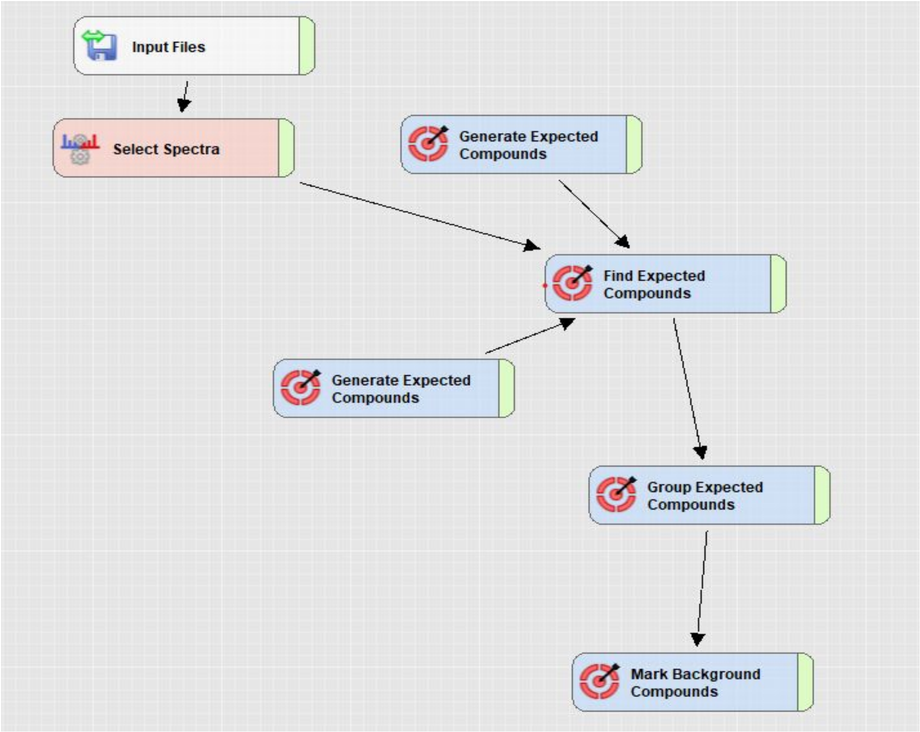

Figure S1: Compound Discoverer workflows used for the Molecular Networking approach (top) and Expected Compounds approach (bottom).

Table S5. Compound Discoverer parameters

**Detect Compounds**		**Generate Expected Compounds**	
Mass Tolerance	5 ppm	Max # Phase I	1
Intensity Tolerance	30	Max # All Steps	3
S/N Threshold	4	Phase 1 (all compounds)	H2 O -><br>H2 -><br>H O -><br>H ->
Min. Peak Intensity	100000	Phase 1 (HMMM)	C H3 -> H
Ions	[M+H]+1;<br>[M+K]+1;<br>[M+Na]+1	Phase 2	See Table S4
**Group Compounds**		**Find Expected Compounds**	
Mass Tolerance	10 ppm	Mass Tolerance	5 ppm
RT Tolerance	0.2 min	Intensity Tolerance	30
Fragment Data Selection	[M+H]+1;<br>[M-H]-1	Intensity Threshold	0.1
**Fill Gaps**		SN Threshold	3
Mass Tolerance	5 ppm	Min # Isotope	2
S/N Threshold	1.5	Min. Peak Intensity	100000
**Normalize Areas**			
Min. QC Coverage	50%		
Max QC Area RSD	30%		
**Mark Background Compounds**			
Max. Sample/Blank	5		
**Generate Molecular Networks**			
Use Full MSn Tree	True		
Match Mass Shift	True		
Match Transformation	True		
Variate Transformation	False		
S/N Threshold	3		
Mass Tolerance	2.5 mmu		
Min. Fragment m/z	50		
Require MS2	True		
Min. MSn Score	20		
Min. MSn Coverage	50		
Min. Matched Fragments	2		

Table S6. Transformations for generating expected compounds

Name	Leaving Group	Arriving Group	ΔM [Da]	Phase	Max Occurrence
Acetamide addition		C2 H5 N O	59.03711	Phase2	1
Acetate addition		C2 H3 O2	59.0133	Phase2	1
Acetylation	H	C2 H3 O	42.01056	Phase2	1
Alanine addition		C3 H7 N O2	89.04768	Phase2	1
AQC derivatization		C10 H6 N2 O	170.048	Phase1	1
Arginine Conjugation	H O	C6 H13 N4 O2	156.1011	Phase2	1
C6H10O3 unknown addition		C6 H10 O3	130.063	Phase2	1
CH2O addition		C H2 O	30.01056	Phase2	1
CH4O addition		C H4 O	32.02621	Phase2	1
CO addition		C O	27.99491	Phase2	1
CO+H2O		C H2 O2	46.00548	Phase2	1
CO2 addition		C O2	43.98983	Phase2	1
Cysteine Conjugation 1	H	C3 H6 N O2 S	119.0041	Phase2	1
Cysteine Conjugation 2		C3 H7 N O2 S	121.0198	Phase2	1
Dehydration	H2 O		-18.0106	Phase1	2
Deoxy hexoside		C6 H10 O4	146.0579	Phase2	1
Desaturation	H2		-2.01565	Phase1	3
Formic acid addition		C H2 O2	46.00548	Phase2	1
Gallic acid addition	H2 O	C7 H6 O5	152.011	Phase2	1
Glucoronic acid addition		C6 H10 O7	194.0427	Phase2	1
Glucose addition		C6 H12 O6	180.0634	Phase2	1
Glucose_glucose		C12 H20 O10	324.1057	Phase2	1
Glucoside Conjugation	H	C6 H11 O5	162.0528	Phase2	1
Glucuronide addition		C6 H8 O6	176.0321	Phase2	1
Glucuronide Conjugation	H	C6 H9 O6	176.0321	Phase2	1
Glutamine Conjugation	H O	C5 H9 N2 O3	128.0586	Phase2	1
Glycine addition		C2 H5 N O2	75.03203	Phase2	1
Glycine Conjugation	H O	C2 H4 N O2	57.02146	Phase2	1
GSH Conjugation 1		C10 H15 N3 O6 S	305.0682	Phase2	1
GSH Conjugation 2		C10 H17 N3 O6 S	307.0838	Phase2	1
H2CN addition		C H2 N	28.01872	Phase2	1
HCN addition		C H N	27.0109	Phase2	1
HMMMtrans_demethylation	C H3	H	-14.0157	Phase1	6
HSO3 addition		H O3 S	80.96464	Phase2	1
Ketene addition		C2 H2 O	42.01056	Phase2	1

acetyl-alanine conjugation	H	C5 H8 N O3	129.0426	Phase2	1
173 conjugation	H	C6 H8 N O5	173.0324	Phase2	1
alanine conjugation	H	C3 H6 N O2	87.03203	Phase2	1
C12H13NO6 addition		C12 H13 N O6	267.0743	Phase2	1
Malonyl + glucose		C9 H12 O8	248.0532	Phase2	1
Malonyl + glucosyl		C8 H10 O7	218.0427	Phase2	1
Malonyl addition		C3 H2 O3	86.00039	Phase2	1
Methylation	H	C H3	14.01565	Phase2	1
N-acetilglucosamina		C8 H13 N O5	203.0794	Phase2	1
N-acetylcysteine		C5 H9 N O3 S	163.0303	Phase2	1
NH3 addition		H3 N	17.02655	Phase2	1
NO addition		N O	29.99799	Phase2	1
NO2 addition		N O2	45.9929	Phase2	1
OH removal	H O		-17.0027	Phase1	1
Ornitine Conjugation	H O	C5 H11 N2 O2	114.0793	Phase2	1
Palmitoyl Conjugation	H	C16 H31 O	238.2297	Phase2	1
Reduction	H		-1.00783	Phase1	2
Stearyl Conjugation	H	C18 H35 O	266.261	Phase2	1
Taurine Conjugation	H O	C2 H5 N O3 S	105.9963	Phase2	1
Unknown addition C5H10N2O3		C5 H10 N2 O3	146.0691	Phase2	1
Unknown addition C7H10O3		C7 H10 O3	142.063	Phase2	1
Unknown C10H17N3O6S		C10 H17 N3 O6 S	307.0838	Phase2	1
Unknown C6H9O8S		C6 H9 O8 S	241.0018	Phase2	1
Unknown C8H11O9S		C8 H11 O9 S	283.0124	Phase2	1
Unknown C8H14N2O5S		C8 H14 N2 O5 S	250.0623	Phase2	1
Unknown C8H15NO6		C8 H15 N O6	221.0899	Phase2	1
Unknown C9H14O9		C9 H14 O9	266.0638	Phase2	1
Xylose addition	H2 O	C5 H10 O5	132.0423	Phase2	1

Table S7. Concentration of TWP-derived compounds per unit biomass in lettuce leaves: Results of Tukey's HSD multiple comparisons across compounds for each time point. Different lowercase letters indicate statistically significant differences between compounds ($p<0.05$).

a) spiked compounds

	DPG	BTZ	HMMM	6PPD	6PPD-q
3 hrs	cd	a	b	d	bc
6 hrs	d	a	b	cd	c
12 hrs	b	b	a	b	b
1 day	b	b	a	b	b
2 days	b	b	a	b	b
4 days	b	b	a	b	b
7 days	b	d	a	cd	bc
10 days	b	c	a	c	c
14 days	b	b	a	b	b

b) tire leachate

	DPG	BTZ	HMMM	6PPD	6PPD-q
3 hrs	a	b	b	b	b
6 hrs	a	c	b	c	c
12 hrs	a	b	a	b	b
1 day	b	c	a	c	c
2 days	b	b	a	b	b
4 days	b	b	a	b	b
7 days	b	b	a	b	b
10 days	b	b	a	b	b
14 days	b	b	a	b	b

Table S8. Concentration of TWP-derived compounds per unit biomass in lettuce leaves: Results of Tukey's HSD multiple comparisons across time points for each compound. Different lowercase letters indicate statistically significant differences between time points (p<0.05).

a)spiked compounds

	3 hrs	6 hrs	12 hrs	1 day	2 days	4 days	7 days	10 days	14 days
DPG	d	cd	d	d	cd	bc	a	ab	b
BTZ	ab	a	cd	bc	cde	def	ef	f	f
HMMM	e	e	e	de	cd	bc	ab	a	abc
6PPD	c	c	bc	bc	bc	b	a	bc	c
6PPD-q	b	ab	ab	ab	b	ab	ab	ab	a

b) tire leachate

	3 hrs	6 hrs	12 hrs	1 day	2 days	4 days	7 days	10 days	14 days
DPG	c	c	c	bc	bc	c	bc	b	a
BTZ	ab	a	a	a	a	a	ab	a	b
HMMM	c	c	c	c	c	bc	b	a	a
6PPD	b	b	b	ab	b	b	a	a	a
6PPD-q	c	c	bc	abc	bc	bc	bc	ab	a

Table S9. Translocation factors of TWP-derived compounds from lettuce roots to the leaves after a single initial compound spike: Results of Tukey's HSD multiple comparisons across compounds for each time point. Different lowercase letters indicate statistically significant differences between compounds (p<0.05). NA indicates insufficient data for statistical comparisons.

	HMMM	BTZ	DPG	6PPD	6PPD-q
3 hrs	a	a	b	b	b
6 hrs	NA	NA	NA	NA	NA
12 hrs	a	a	b	b	b
1 day	a	b	b	b	b
2 days	ab	ab	b	b	b
4 days	a	b	b	b	b
7 days	a	b	b	b	b
10 days	a	b	b	b	b
14 days	a	b	b	b	b

Table S10. Ion intensities of TWP-derived compounds and their transformation products over time: Results of Tukey's HSD multiple comparisons across compounds for each time point. Different lowercase letters indicate statistically significant differences between compounds (p<0.05).

	3 hrs	6 hrs	12 hrs	1 day	2 days	4 days	7 days	10 days	14 days
**DPG**	d	cd	d	d	d	bcd	abc	a	ab
$TP_{DPG}266$	b	ab	b	b	b	b	ab	ab	a
$TP_{DPG}270$	c	bc	c	c	c	c	bc	a	ab
$TP_{DPG}268$	b	b	b	b	b	b	a	a	a
**BTZ**	ab	ab	ab	ab	b	ab	ab	a	ab
$TP_{BTZ}152$	c	bc	c	c	c	bc	b	a	a
**HMMM**	d	cd	cd	cd	bc	b	b	a	a
$TP_{HMMM}271$	a	a	a	a	a	a	a	a	a
$TP_{HMMM}377$	d	cd	d	d	bcd	bc	b	a	a
$TP_{HMMM}363$	c	bc	bc	bc	bc	bc	b	a	a
$TP_{HMMM}359$	e	cde	de	cde	bcd	bc	b	a	a
$TP_{HMMM}345$	d	cd	d	cd	bcd	bc	b	a	a
$TP_{HMMM}331$	b	b	b	b	b	b	b	a	a
$TP_{HMMM}301$	b	b	b	b	b	b	b	a	a
$TP_{HMMM}303$	b	b	b	b	b	b	b	a	a
$TP_{HMMM}546$	a	a	a	a	a	a	a	a	a
**6PPD**	c	bc	bc	bc	bc	b	a	bc	bc
$TP_{6PPD}431$	c	abc	bc	ab	a	a	a	abc	c
**6PPD-q**	b	b	b	b	b	b	b	b	a
$TP_{6PPD\text{-}q}262$	a	a	a	a	a	a	a	a	a
$TP_{6PPD\text{-}q}174$	a	bc	b	bcd	cde	de	ef	ef	f
$TP_{6PPD\text{-}q}214$	a	a	a	a	a	a	a	a	a

Table S11. Concentrations of TWP-derived compounds in the nutrient solution with and without lettuce plants: Results of ANOVA testing for each compound and time point. Asterisk (*) indicates statistically significant differences between samples with and without lettuce plants (p<0.05).

a) spiked compounds

	0	3 hrs	6 hrs	12 hrs	1 day	2 days	4 days	7 days	10 days	14 days
DPG			*	*	*	*	*	*	*	*
BTZ				*	*	*	*	*	*	*
HMMM				*					*	*
6PPD				*	*	*	*	*		*
6PPD-q		*	*	*	*	*	*	*	*	*

b) tire leachate

	3 hrs	6 hrs	12 hrs	1 day	2 days	4 days	7 days	10 days	14 days
DPG	*		*						*
BTZ			*	*	*	*	*	*	*
HMMM	*	*				*			
6PPD	*	*		*	*			*	
6PPD-q	*			*			*		

Table S12. Biomass of lettuce plants at the beginning and end of the experiment.

		Plants exposed to TWP				Plants exposed to initial spike			
		plant weight [g]		weight difference		plant weight [g]		weight difference	
time point	*plant*	*beginning*	*end*	*total*	*%*	*beginning*	*end*	*total*	*%*
1	A	1.95	1.86	-0.09	-4.56	1.50	1.49	-0.01	-0.8
1	B	2.62	2.57	-0.06	-2.10	0.89	0.86	-0.03	-3.5
1	C	2.02	2.01	-0.01	-0.54	1.20	1.17	-0.03	-2.9
2	A	1.99	2.01	0.02	1.11	1.41	1.28	-0.13	-9.4*
2	B	1.29	1.28	-0.01	-0.78	1.49	1.41	-0.08	-5.5*
2	C	1.38	1.34	-0.04	-2.83	2.11	2.09	-0.02	-1.1
3	A	2.12	2.10	-0.02	-0.80	1.89	1.90	0.01	0.3
3	B	1.37	1.35	-0.02	-1.61	1.54	1.49	-0.05	-3.2
3	C	1.68	1.72	0.04	2.08	1.35	1.30	-0.05	-3.9
4	A	1.07	1.07	0.00	-0.09	2.05	2.02	-0.03	-1.4
4	B	1.45	1.44	-0.01	-0.76	1.09	1.06	-0.03	-3.0
4	C	0.95	0.92	-0.03	-3.16	1.45	1.41	-0.04	-2.6
5	A	2.76	2.71	-0.05	-1.74	1.16	1.21	0.05	4.2
5	B	1.47	1.50	0.03	2.11	1.17	1.12	-0.05	-4.5
5	C	1.50	1.48	-0.02	-1.07	1.26	1.28	0.01	1.2
6	A	2.04	N/A	N/A	N/A	1.52	2.17	0.65	42.6
6	B	2.84	N/A	N/A	N/A	0.72	0.70	-0.02	-2.2
6	C	1.9	N/A	N/A	N/A	1.60	2.13	0.53	33.2
7	A	2.13	2.39	0.26	12.30	1.11	1.26	0.15	13.2
7	B	1.24	1.36	0.12	9.44	1.16	0.83	-0.33	-28.3*
7	C	0.91	0.88	-0.03	-3.30	1.145	1.25	0.10	8.9
8	A	2.98	3.34	0.36	12.01	1.64	2.07	0.43	26.2
8	B	2.20	2.55	0.35	15.77	1.81	2.27	0.46	25.6
8	C	0.86	1.02	0.16	18.84	1.55	1.10	-0.45	-28.9*
9	A	2.53	3.12	0.59	23.20	1.60	1.86	0.26	16.1
9	B	1.94	2.13	0.19	9.74	1.51	2.01	0.50	32.8
9	C	1.68	1.88	0.20	12.08	1.37	1.67	0.30	21.6

Plants with biomass loss > 5% (marked with an asterisk) were excluded from further analysis.

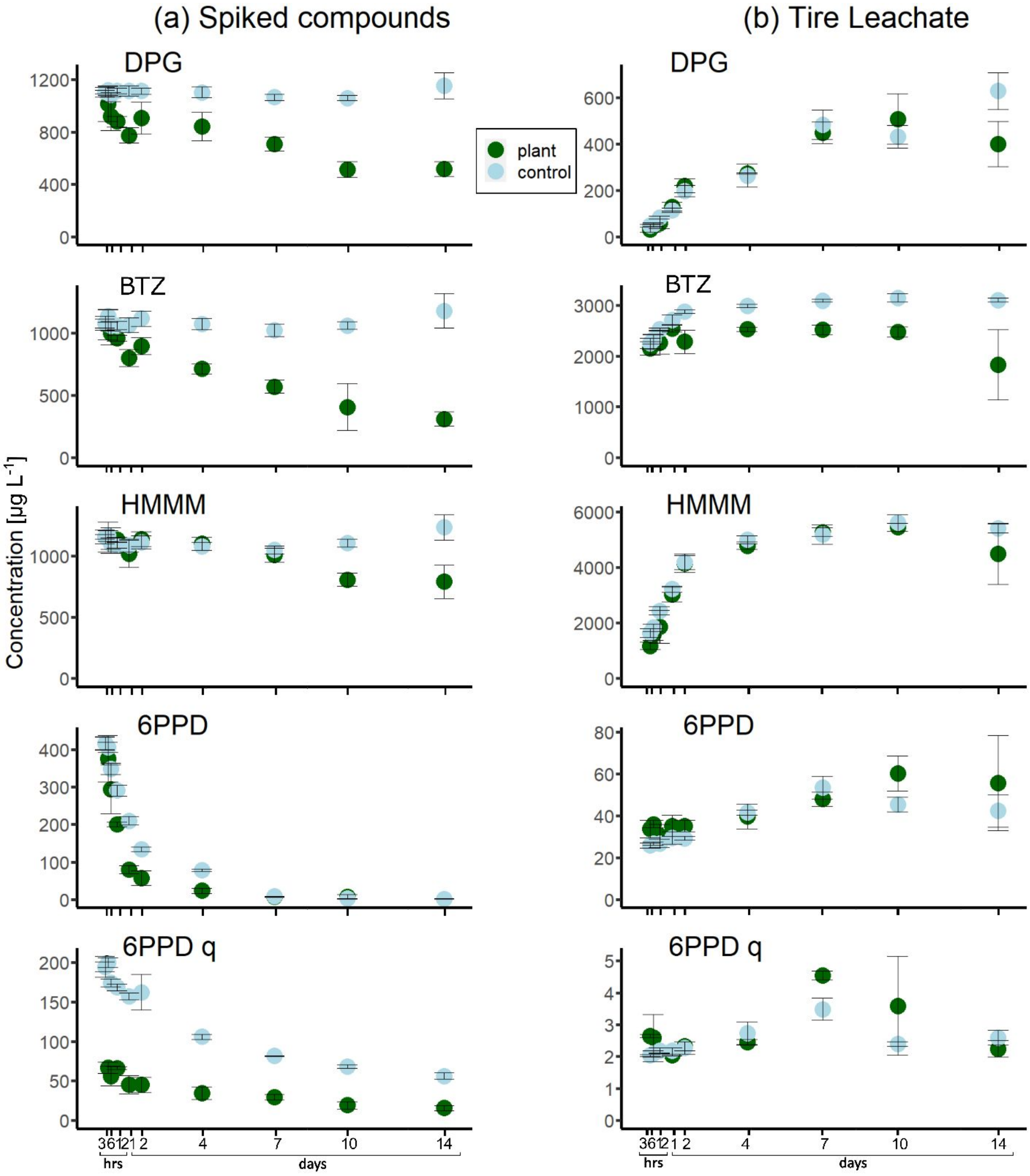

Figure S2. Concentrations of TWP-derived compounds in the nutrient solution with lettuce plants (● green dots) or without plants (● blue dots) over 14 days after exposure to (a) a single initial TWP-derived compound spike and (b) continuously replenished leaching from TWP in the nutrient solution. Error bars represent the standard deviation from triplicate measurements.

Table S13. K_D values of TWP-derived compounds in lettuce root tissue at initial aqueous concentrations of 50 and 500 μgL^{-1}.

Initial concentration	DPG	BTZ	HMMM	6PPD	6PPD-q
50 μg/L	0.68	0.06	0.10	0.26	N/A
500 μg/L	0.17	0.05	0.05	1.99	N/A

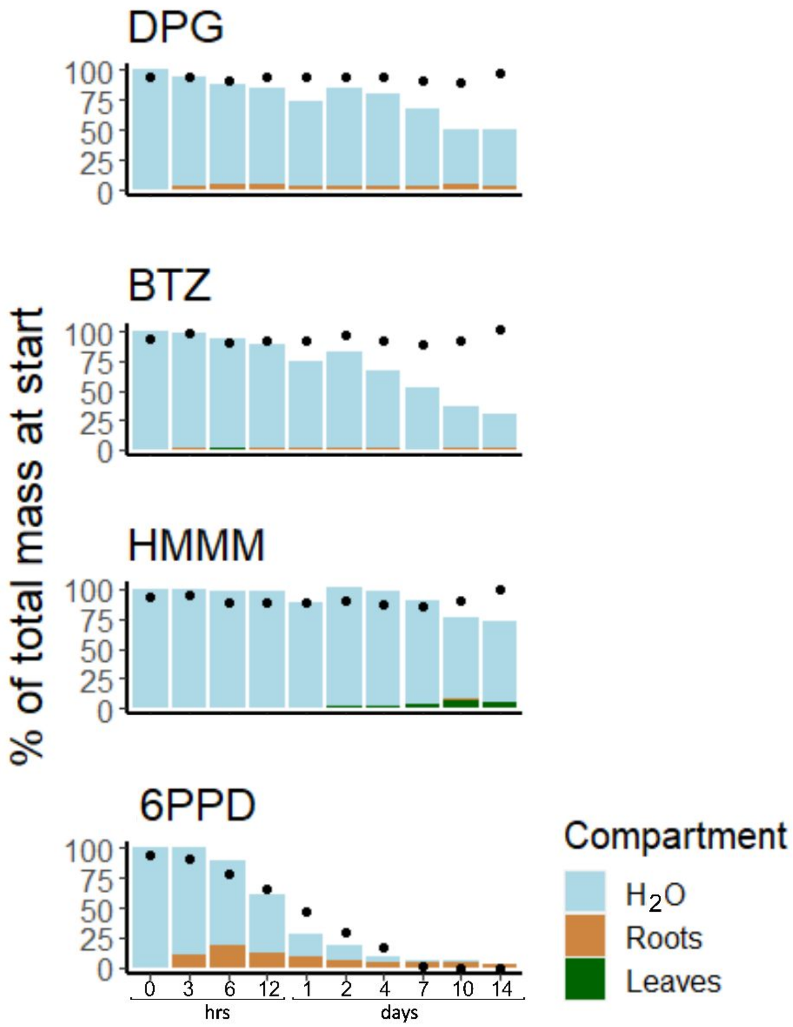

Figure S3. Mass of DPG, BTZ, HMMM and 6PPD in the nutrient solution (blue bars ■), roots (brown bars ■) and in lettuce leaves (green bars ■) relative to the total mass added at the start. Abiotic controls without plants are represented as black dots (●).

Section S4. Transformation products of TWP-derived compounds

Information for all transformation products, arranged by TWP-derived compounds, are shown in Table S7. The naming scheme for the transformation products was adapted from Alhelou et al[1]. All molecular formulas proposed are based on a mass error of less than 5PPM. Fragments marked with an asterisk (*) were used as diagnostic fragments to confirm either a compound's structure, or its relation to its parent compound. Diagnostic fragments are either exact fragment matches, or fragment matches adjusted by the calculated mass shift between the transformation product and the parent compound. Exact fragment matches are based on our measurements or on literature (in this case, appropriate studies are cited). In addition to reporting diagnostic fragments, we report all fragments measured on the Orbitrap-HRMS at an intensity level > $5x10^4$. For many transformation products, we do not propose exact structures but rather highlight the relevant reaction components (e.g. because we were unable to determine the exact site of conjugation). Confidence levels were adapted from Schymanski et al[2] as follows:

1: *Confirmed Structure* with Reference Standard. MS, MS^2, RT matching.
2(a): *Probable Structure* with Library matches. MS, MS^2 matching to literature
3: Tentative Candidates: MS supports probable reaction. MS^2 confirm relation to parent compound.
4: Unequivocal Molecular Formula: No structure proposed. Formula based on exact mass, MS^2 confirms relation to parent compound.
5: Exact mass of interest. More than one possible molecular formula. MS^2 confirms relation to parent compound.

Table S14. Transformation products of TWP-derived compounds

ID	Monisotopic Mass	Δ ppm	Proposed Molecular Formula	Proposed Structure/Reactions	Assigned Confidence Level	RT	Fragments
**Benzothiazole**							
BTZ	135.01403	1.78	C_7H_5NS	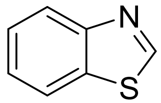	Parent	5.2	72.02843; 83.53615; 136.02126
$TP_{BTZ}152$	151.00888	2.02	C_7H_5NOS	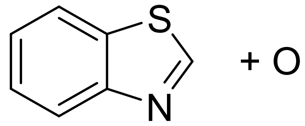	3	3.9	88.02333; 99.5122; 97.02833*; 99.53137*; 152.01601
**6PPD**							
6PPD	268.19311	3.13	$C_{18}H_{24}N_2$	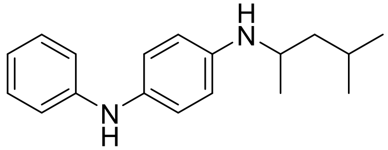	Parent	6.6	93.05740; 184.09885; 185.10664; 269.19952
$TP_{6PPD}431$	430.24539	3.21	$C_{24}H_{34}N_2O_5$	Glucose Conjugation 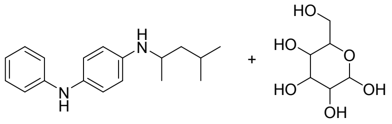	3	5.3	184.09885*; 185.10664;* 209.10664; 268.19205*; 347.15994*; 346.15103*; 431.25165
**6PPD-quinone**							

6PPD-q	298.16702	3.72	$C_{18}H_{22}N_2O_2$	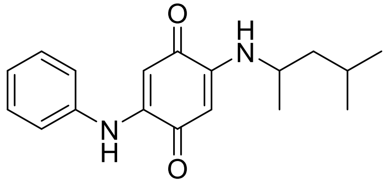	Parent	8.9	100.11225; 187.08591; 215.08073; 241.09608; 243.11143; 256.11960; 299.17422
TP$_{6PPD-q}$262	261.13555	1.53 3.61	$C_{13}H_{17}N_4O_2$ $C_{15}H_{19}NO_3$		5	9.5	206.07991*; 262.14294
TP$_{6PPD-q}$174	173.12001	2.54	$C_{12}H_{15}N$		4	9.4	118.06526*; 132.08080*
TP$_{6PPD-q}$214	213.20858	3.09	$C_{11}H_{25}N_4$ $C_{13}H_{27}NO$		5	9.6	57.07044*
				**HMMM**			
HMMM	390.22129	3.57	$C_{15}H_{30}N_6O_6$	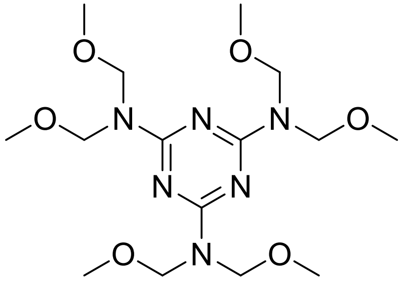	Parent	6.2	72.90399; 107.08781; 159.66292; 167.88551; 176.18416; 177.08781; 192.54492; 193.11816; 207.09831; 253.14046; 283.15042; 301.16147; 329.19138; 359.19931; 373.19775
TP$_{HMMM}$377	376.20581	3.25	$C_{14}H_{28}N_6O_6$	Methoxy hydrolysis	2a	5.5	163.07214[1,3]*; 177.08786[1,3]*; 207.09836[1,3]*;

				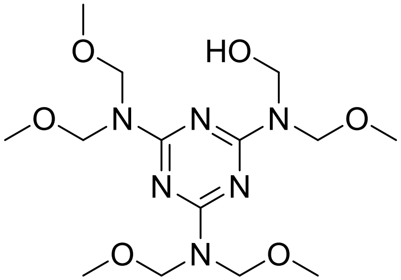			225.76080; 239.12424[1,3]*; 253.13982*; 269.13498[1,3] *; 283.15057[1,3]*; 315.17664
TP$_{HMMM}$363_1	362.19018	3.32	$C_{13}H_{26}N_6O_6$	2x Methoxy hydrolysis 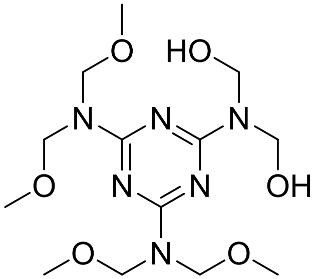	2a	4.9	163.07213*[1,3]; 177.08772*[1,3]; 207.09824*[1,3]; 239.12411*[1,3]; 253.14032*; 255.11877*[1,3]; 269.13443; 283.15106*[1,3]
TP$_{HMMM}$363 isomer	362.19018	3.32	$C_{13}H_{26}N_6O_6$	2x Methoxy hydrolysis 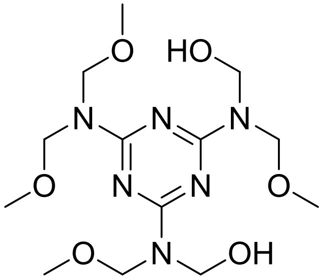	2a	5.5	163.07211*[1,3]; 177.08775*[1,3]; 193.08316*[3]; 207.09827*[1,3]; 225.10896*[3]; 239.12392*[1,3] 253.14059*; 269.13474*[3]; 283.14984*
TP$_{HMMM}$359	358.19526	3.37	$C_{14}H_{26}N_6O_5$	Methanol loss 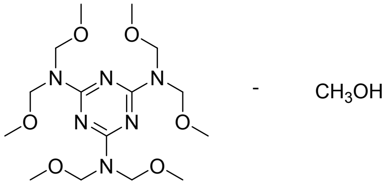	3	6.2	69.75610; 75.14574; 77.06249; 177.08778*; 193.11906*; 207.09814*; 218.30775;

							249.23236; 253.13989*; 283.15073*
TP_{HMMM}345	344.17975	3.10	$C_{13}H_{24}N_6O_5$	Methanol loss and methoxy hydrolysis 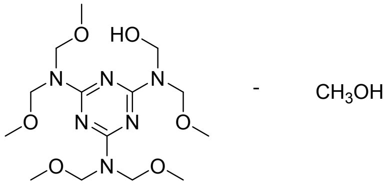	3	5.5	163.07211*; 177.08771*; 207.09827*; 253.14014*; 283.15060*; 163.07211*
TP_{HMMM}345 isomer	344.17975	3.10	$C_{13}H_{24}N_6O_5$	Methanol loss and methoxy hydrolysis 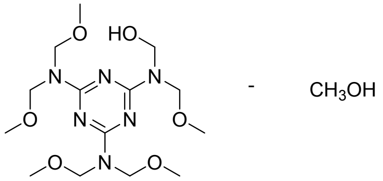	3	6.2	163.07220*; 177.08781*; 207.09836*; 253.14020*; 283.15073*; 163.07220*
TP_{HMMM}331	330.16416	3.05	$C_{12}H_{22}N_6O_5$	Methoxy hydrolysis of TP_{HMMM}345	3	4.9	163.07211*; 177.08774*; 207.09825*; 253.14140*; 163.07211*
TP_{HMMM}301	300.1537	3.01<br>1.45<br>1.46	$C_{11}H_{20}N_6O_4$<br>$C_{10}H_{24}N_2O_8$<br>$C_9H_{18}N_9O_3$		5	4.8	163.07201*; 165.08778; 177.08760*; 195.09839; 207.09785*; 225.10893;

$TP_{HMMM}303$ (Tetra-MMM)	302.16928	3.22	$C_{11}H_{22}N_6O_4$	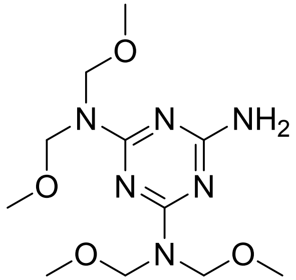	2a	4.5	165.08766*[1,4]; 195.09811*[1,4]; 241.13931*[1]; 271.15005*[1,4];
$TP_{HMMM}271$	270.14313	3.36	$C_{10}H_{18}N_6O_3$	Methanol loss from TetraMMM 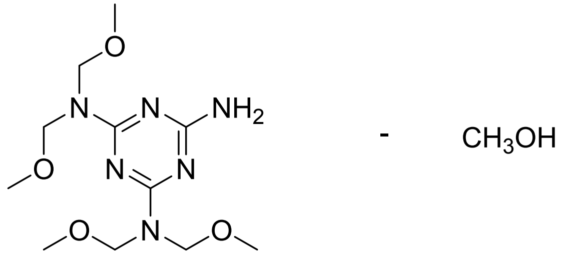	3	4.5	165.08775*; 195.09837*; 241.13919*; 271.1504*
$TP_{HMMM}546$	545.31827	4.22	$C_{21}H_{41}N_{10}O_7$	Reduction (-H), Arginine Conjugation 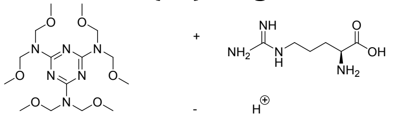	3	8.7	107.08588*; 177.12720*
**DPG**							
DPG	211.11034	2.88	$C_{13}H_{13}N_3$	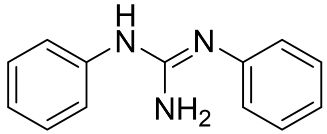	Parent	4.0	94.06531; 119.06019; 195.09102
$TP_{DPG}266$	265.12065	3.25	$C_{16}H_{15}N_3O$		4	4.0	195.09077*
$TP_{DPG}268$	267.09992	3.21	$C_{15}H_{13}N_3O_2$		4	2.3	94.06536*; 119.06019*; 122.05982; 195.09108*; 212.11693*
$TP_{DPG}270$	269.11550	3.44	$C_{15}H_{15}N_3O_2$	-H, acetate addition	3	2.2	119.06023*; 195.09142*

				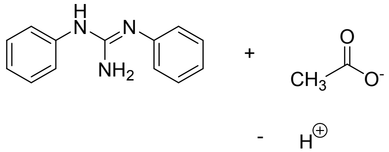				

1. Alhelou, R., Seiwert, B. & Reemtsma, T. Hexamethoxymethylmelamine – A precursor of persistent and mobile contaminants in municipal wastewater and the water cycle. *Water Res.* **165**, 114973 (2019).
2. Schymanski, E. L.; Jeon, J.; Gulde, R.; Fenner, K.; Ruff, M.; Singer, H. P.; Hollender, J. Identifying small molecules via high resolution mass spectrometry: Communicating confidence. *Environ. Sci. Technol.* **48**, 2097–2098 (2014).
3. Wiener, E. A. & Lefevre, G. H. White Rot Fungi Produce Novel Tire Wear Compound Metabolites and Reveal Underappreciated Amino Acid Conjugation Pathways. (2022).
4. Johannessen, C., Helm, P. & Metcalfe, C. D. Detection of selected tire wear compounds in urban receiving waters. *Environ. Pollut.* 135907 (2021) doi:10.1016/j.envpol.2021.117659.

A) BTZ

BTZ MS1 Spectrum

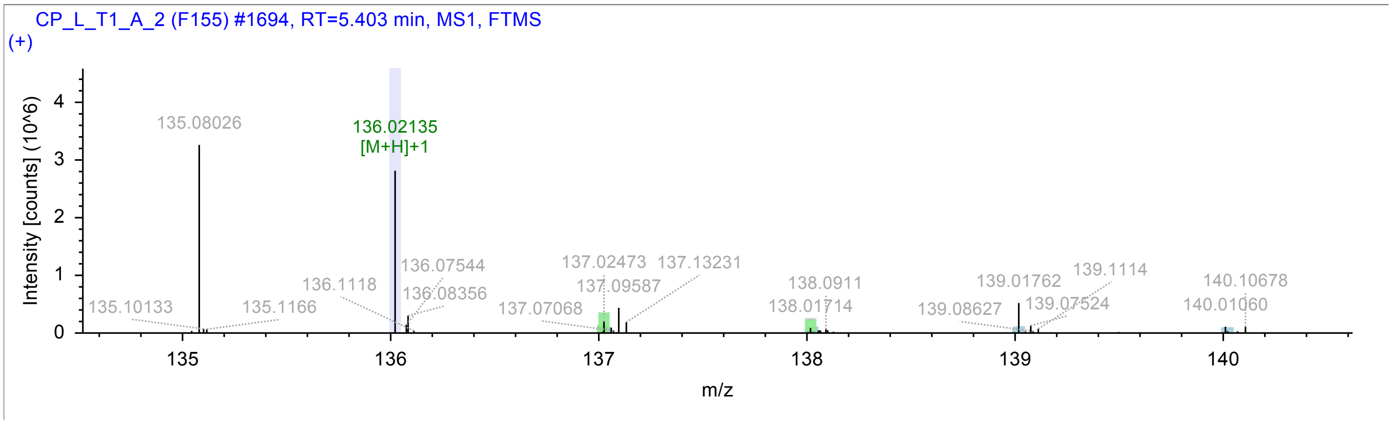

BTZ MS2 Spectrum

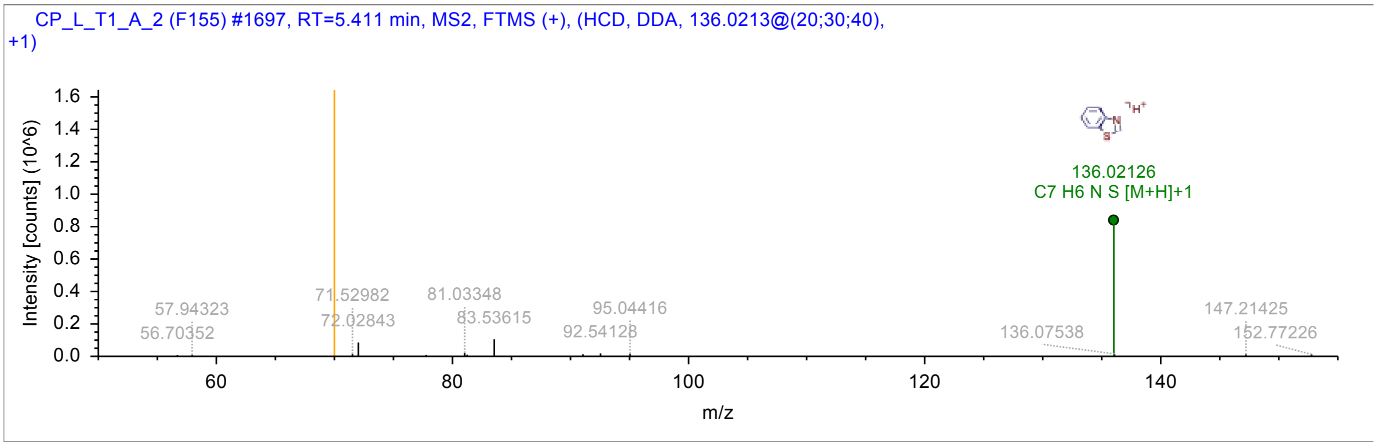

BTZ TP_{BTZ}152 MS1 Spectrum

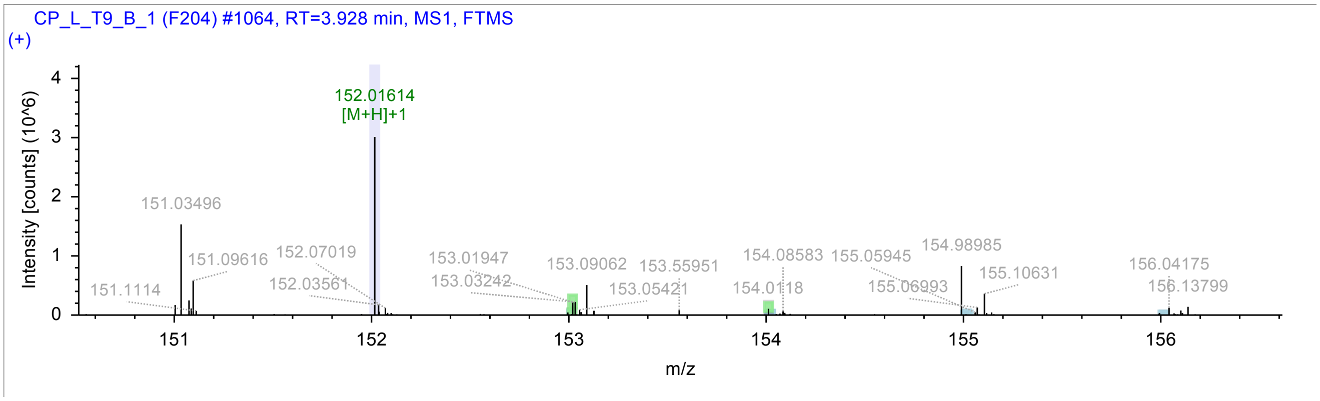

BTZ TP_{BTZ}152 MS2 Spectrum

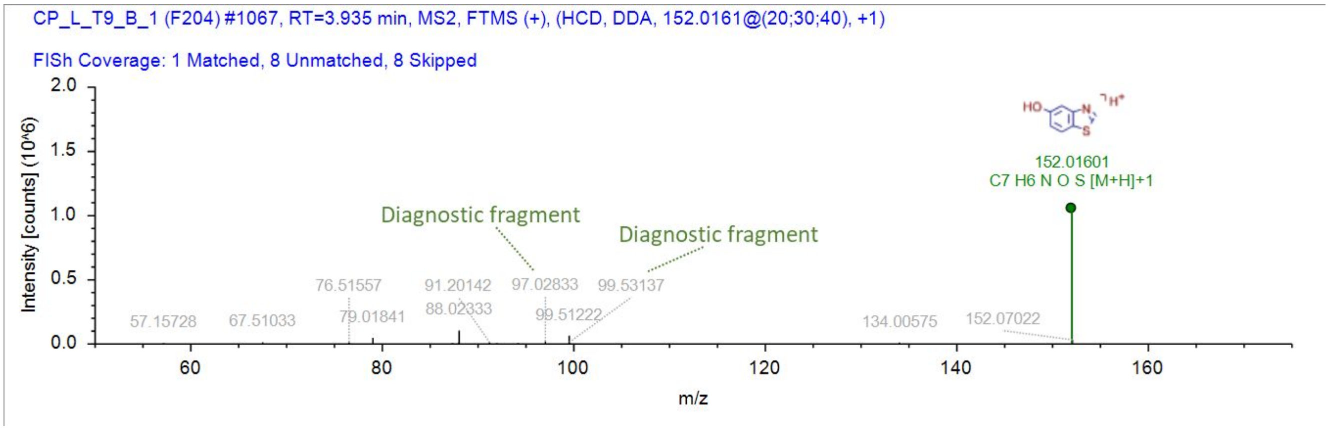

B) 6PPD

6PPD MS1 Spectrum

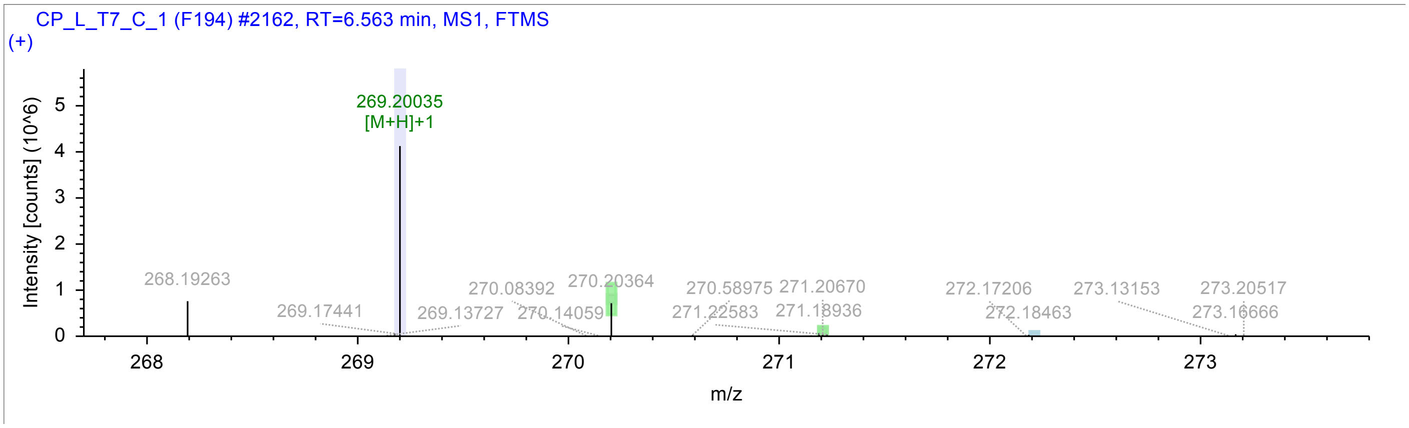

6PPD MS2 Spectrum

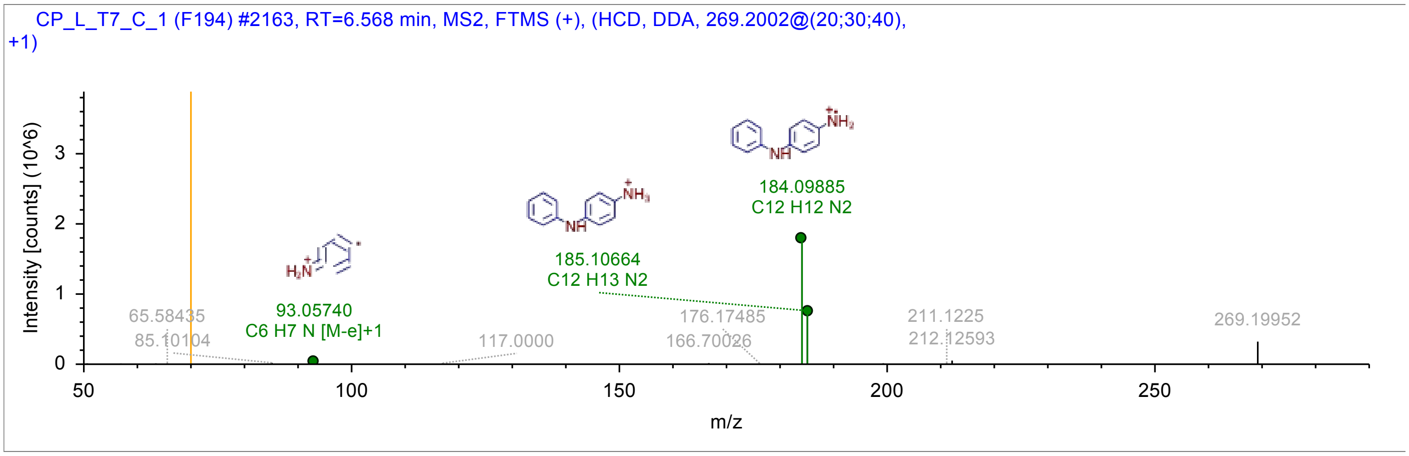

6PPD TP_{6PPD}431 MS1 Spectrum

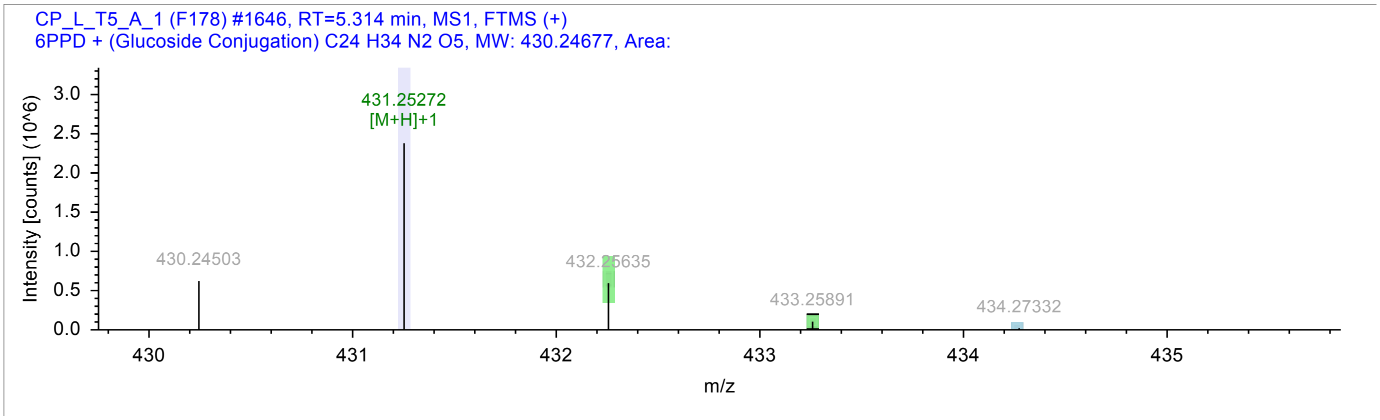

6PPD TP_{6PPD}431 MS2 Spectrum

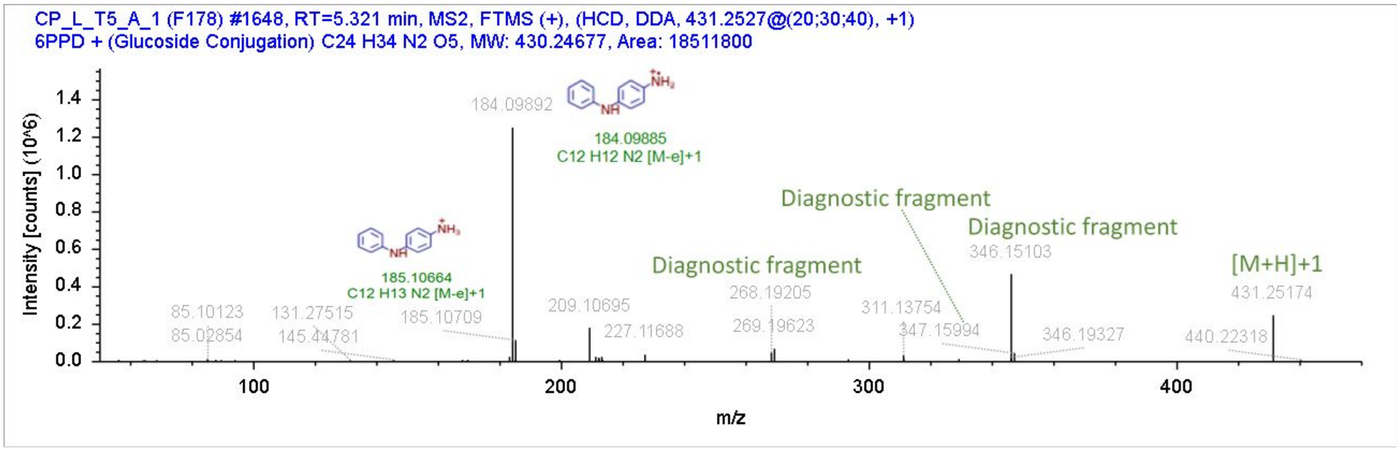

C) 6PPD-q

6PPD-q MS1 Spectrum

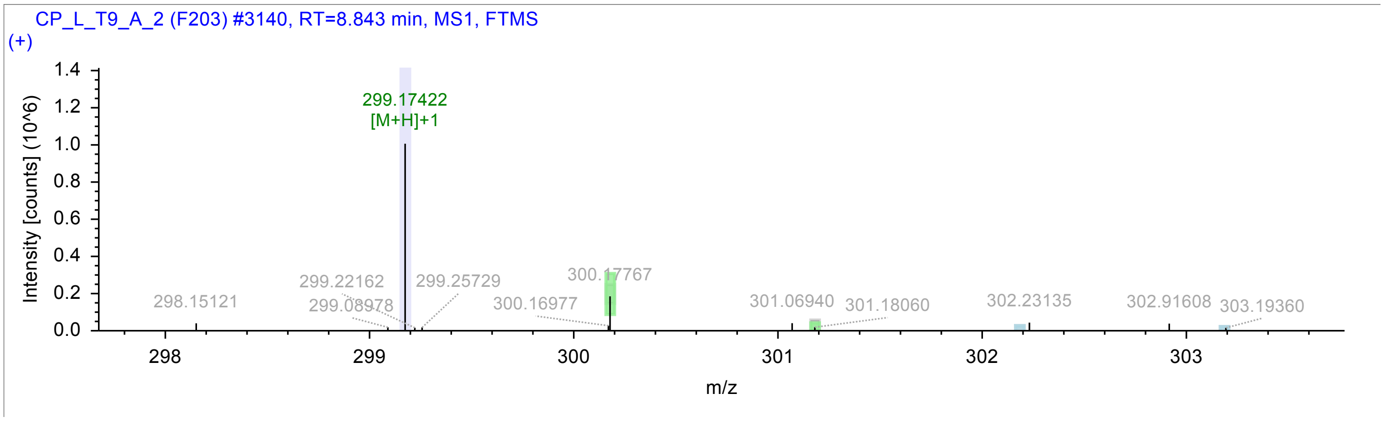

6PPD-q MS2 Spectrum

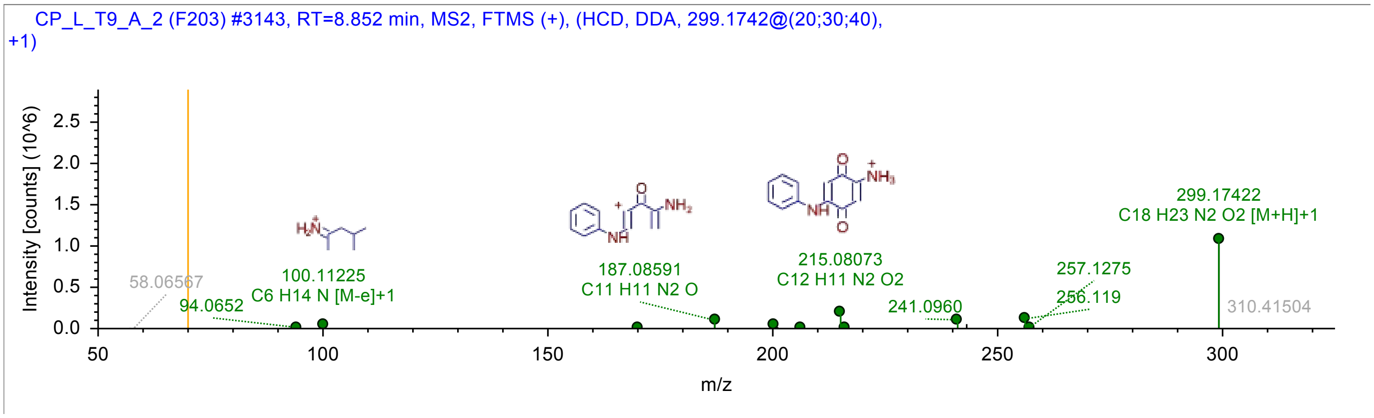

6PPD-q $TP_{6PPD\text{-}q}262$ MS1 Spectrum

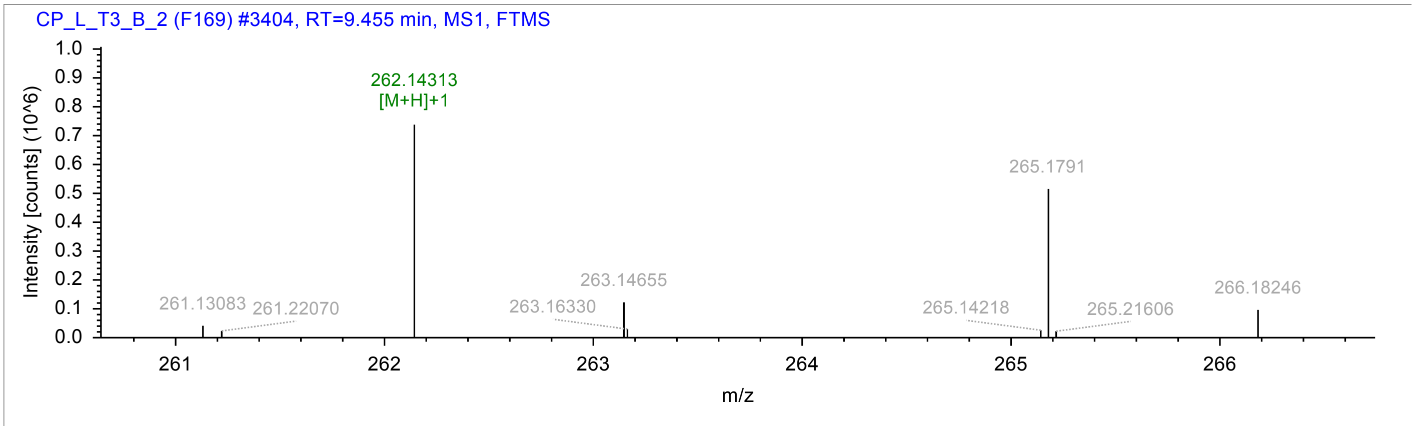

6PPD-q $TP_{6PPD\text{-}q}262$ MS2 Spectrum

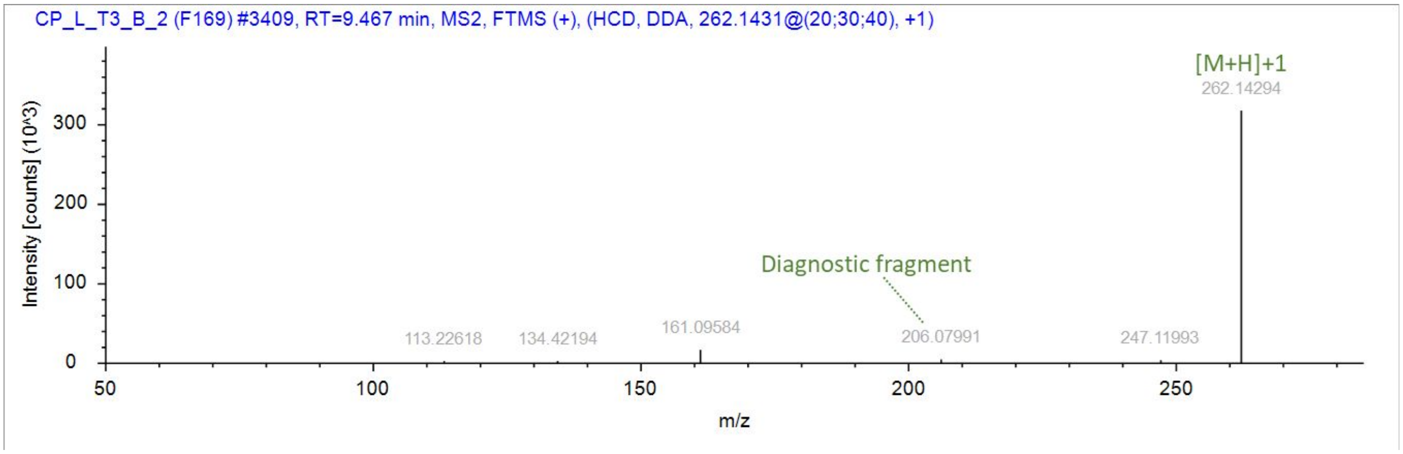

6PPD-q $TP_{6PPD\text{-}q}174$ MS1 Spectrum

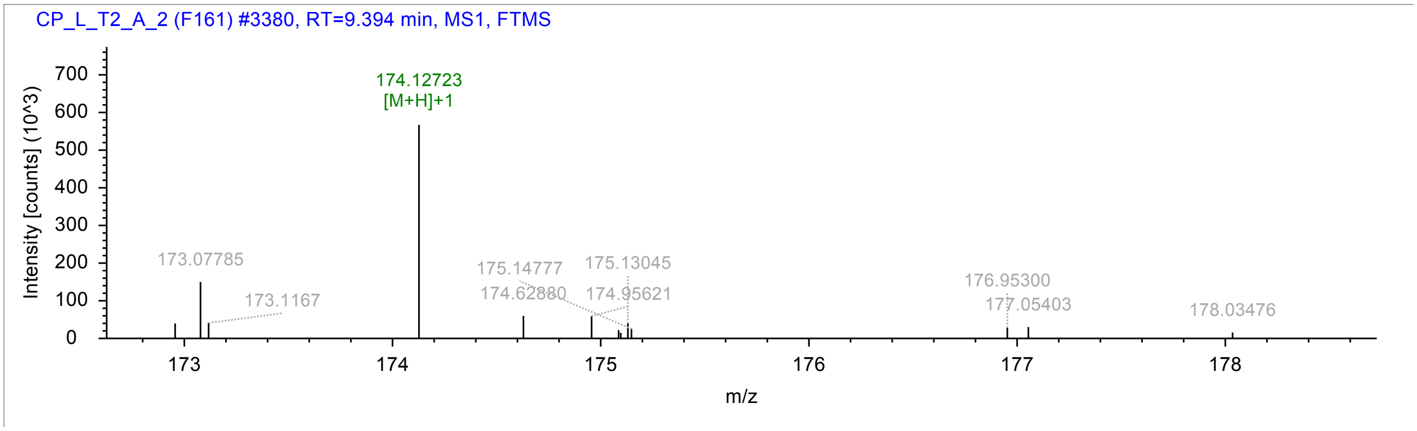

6PPD-q $TP_{6PPD\text{-}q}174$ MS2 Spectrum

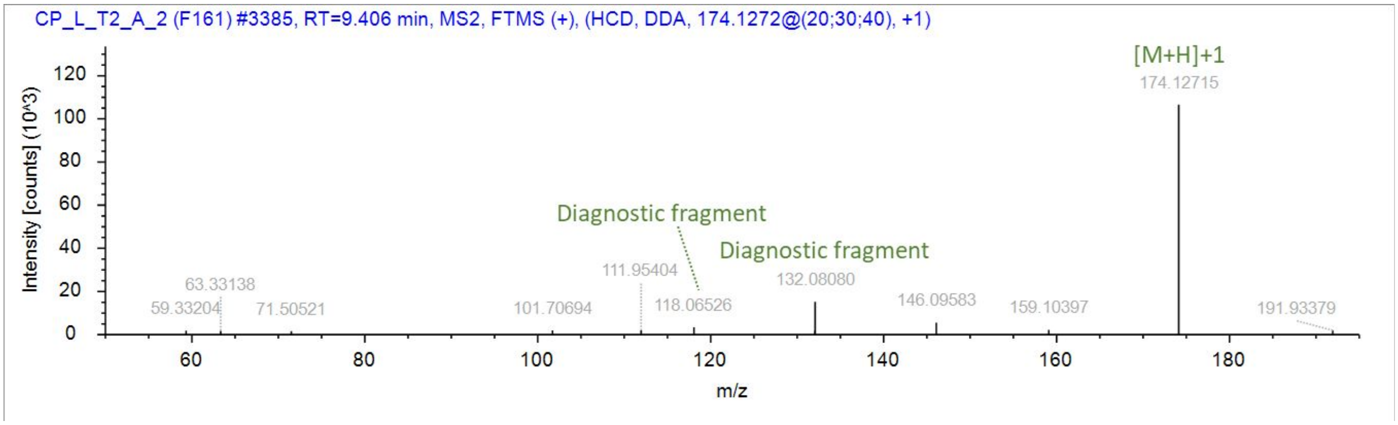

6PPD-q $TP_{6PPD\text{-}q}214$ MS1 Spectrum

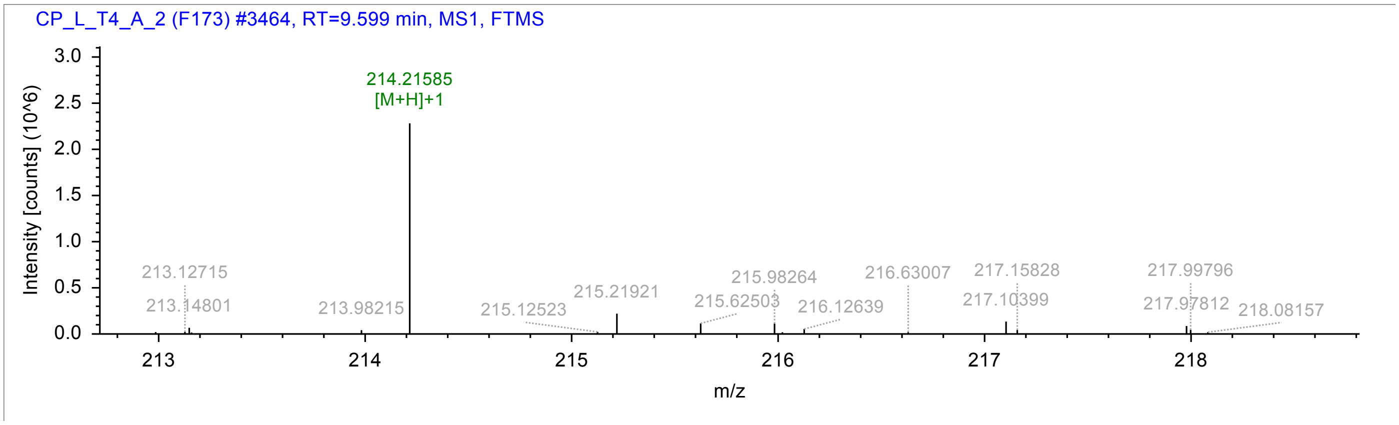

6PPD-q $TP_{6PPD\text{-}q}214$ MS2 Spectrum

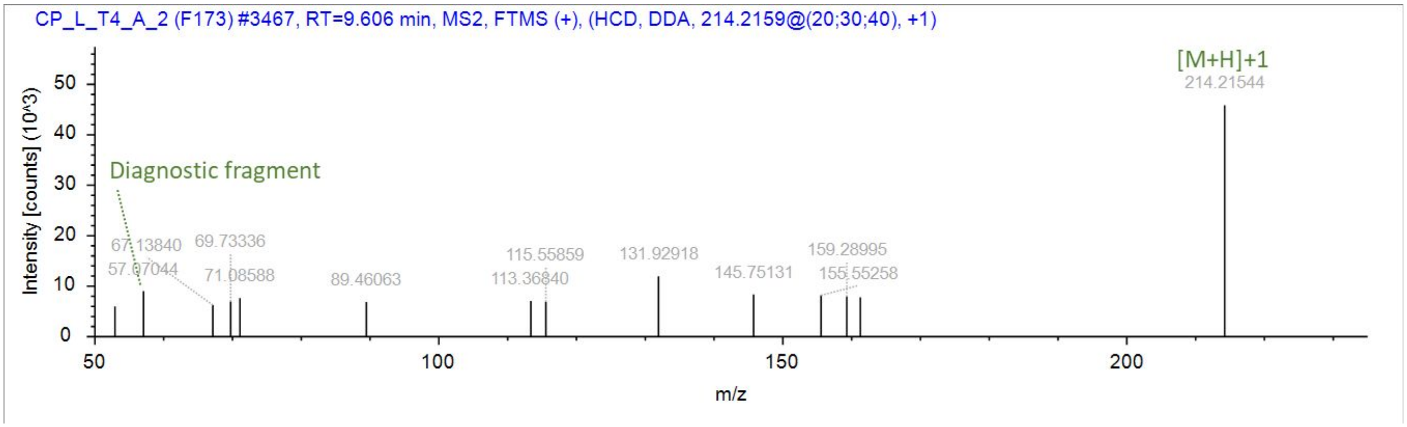

D) HMMM

HMMM MS1 Spectrum

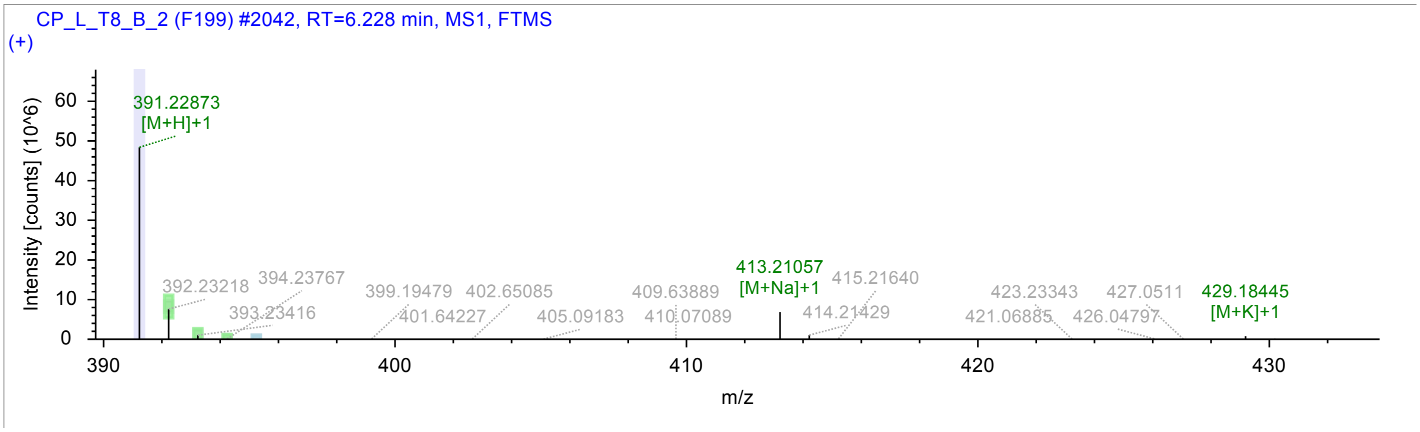

HMMM MS2 Spectrum

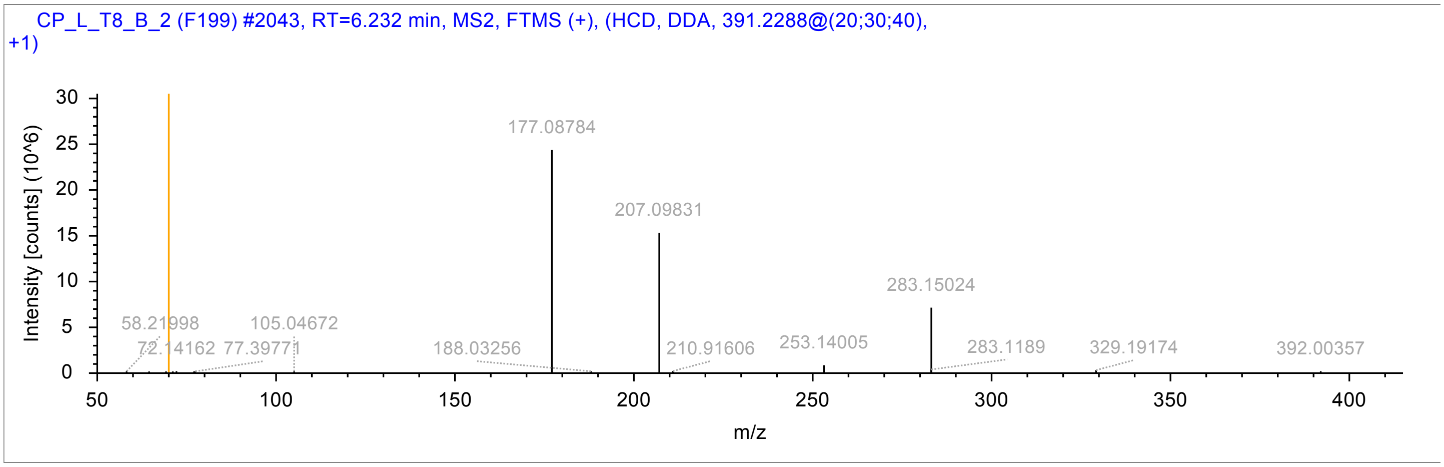

HMMM TP_{HMMM}377 MS1 Spectrum

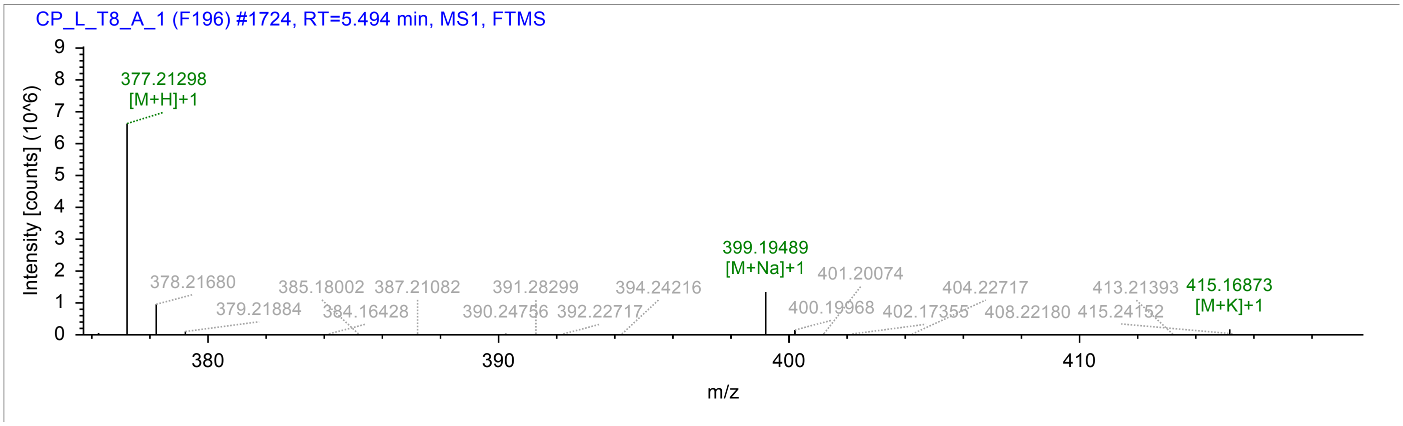

HMMM TP_{HMMM}377 MS2 Spectrum

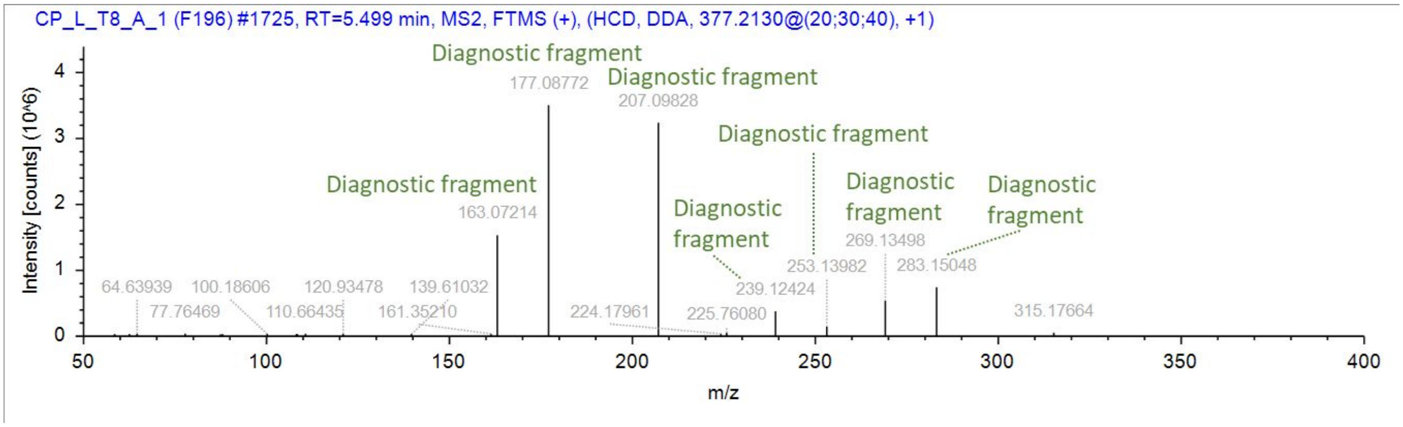

HMMM TP_{HMMM}363_1 MS1 Spectrum

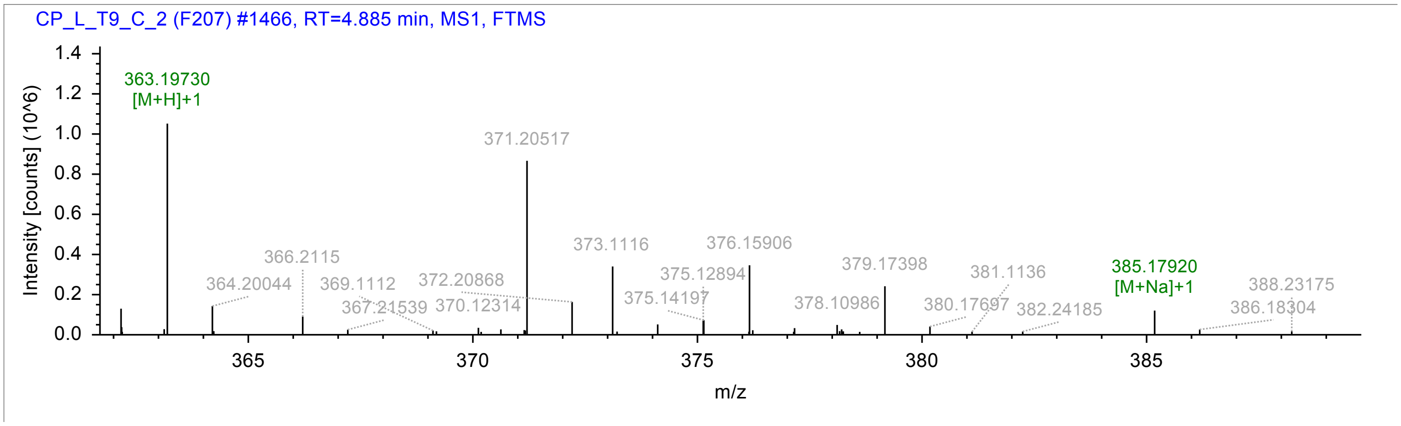

HMMM TP_{HMMM}363_1 MS2 Spectrum

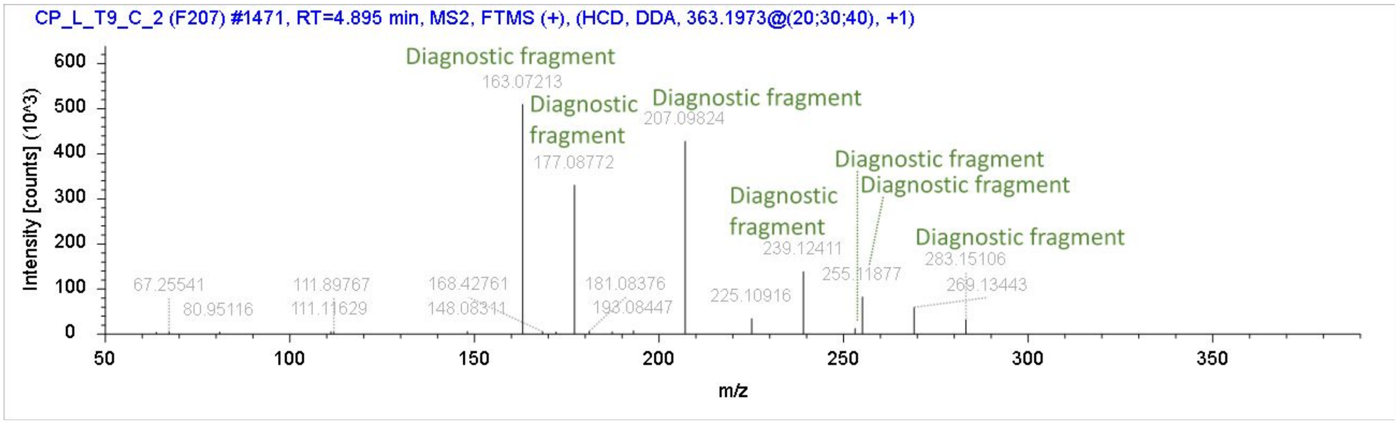

HMMM TP$_{HMMM}$363 isomer MS1 Spectrum

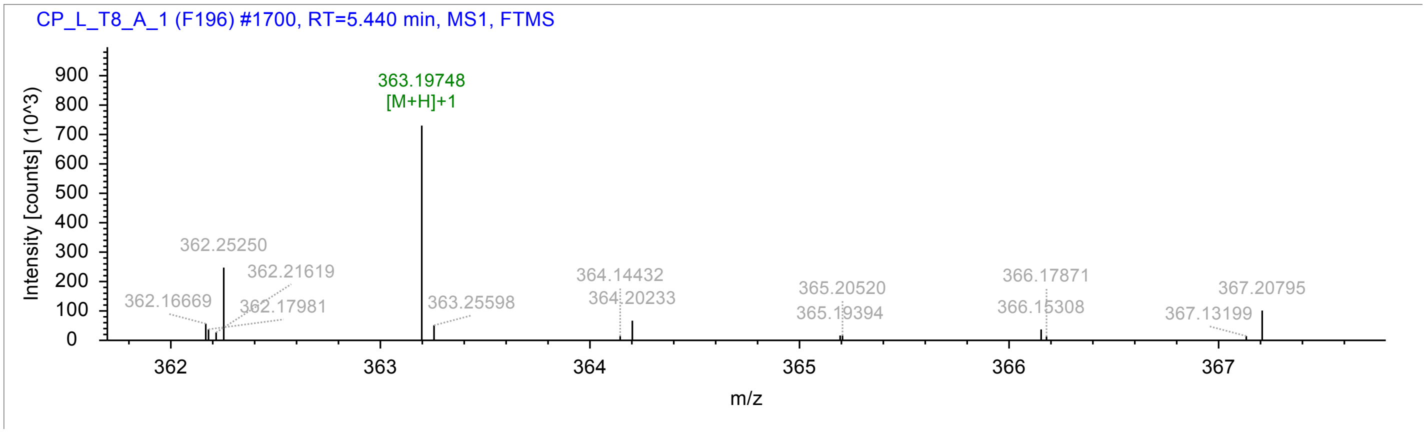

HMMM TP$_{HMMM}$363 isomer MS2 Spectrum

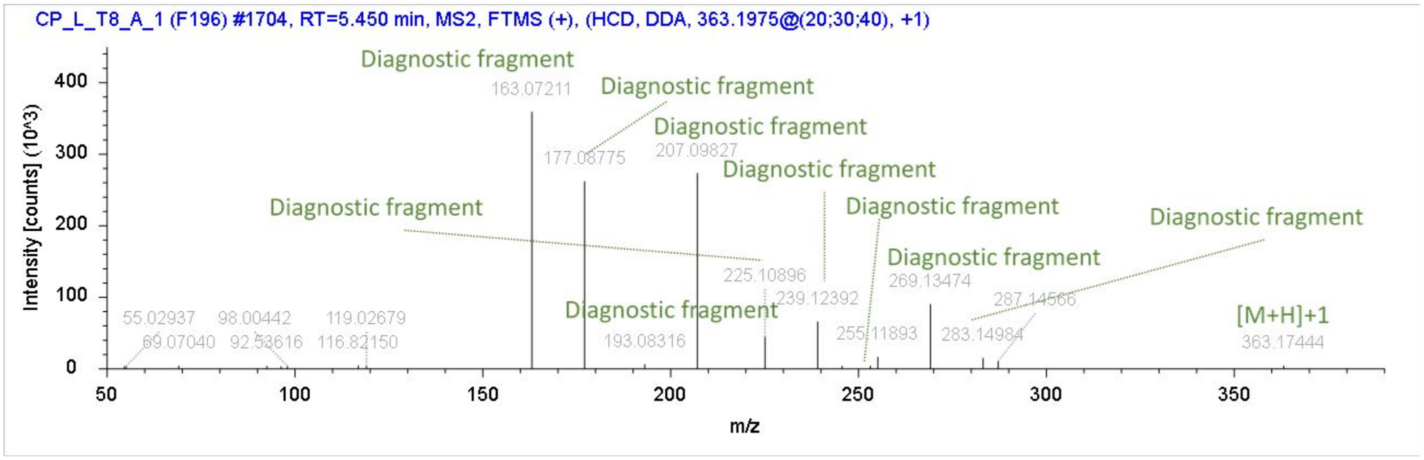

HMMM $TP_{HMMM}359$ MS1 Spectrum

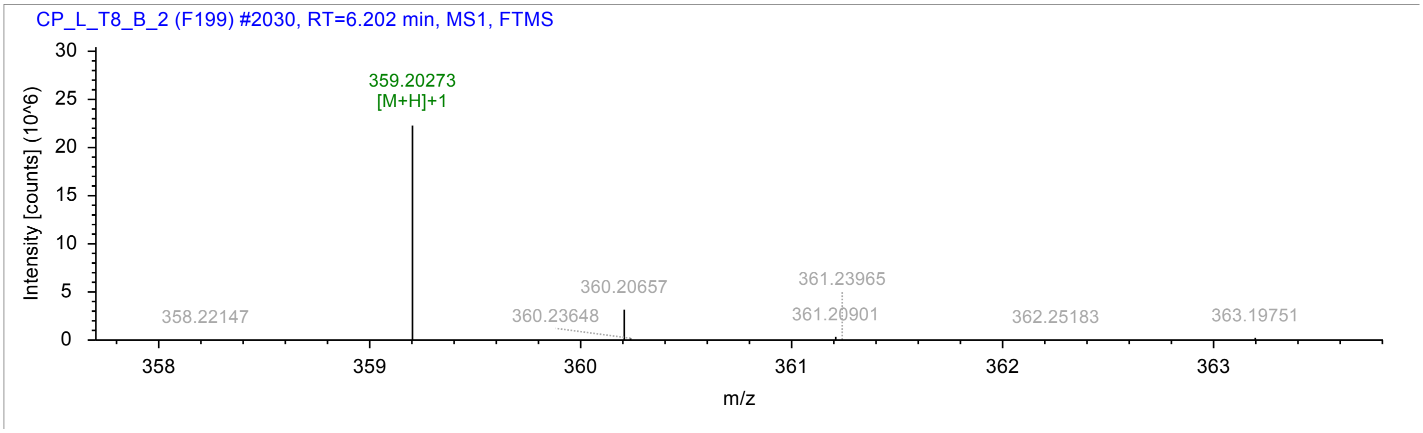

HMMM $TP_{HMMM}359$ MS2 Spectrum

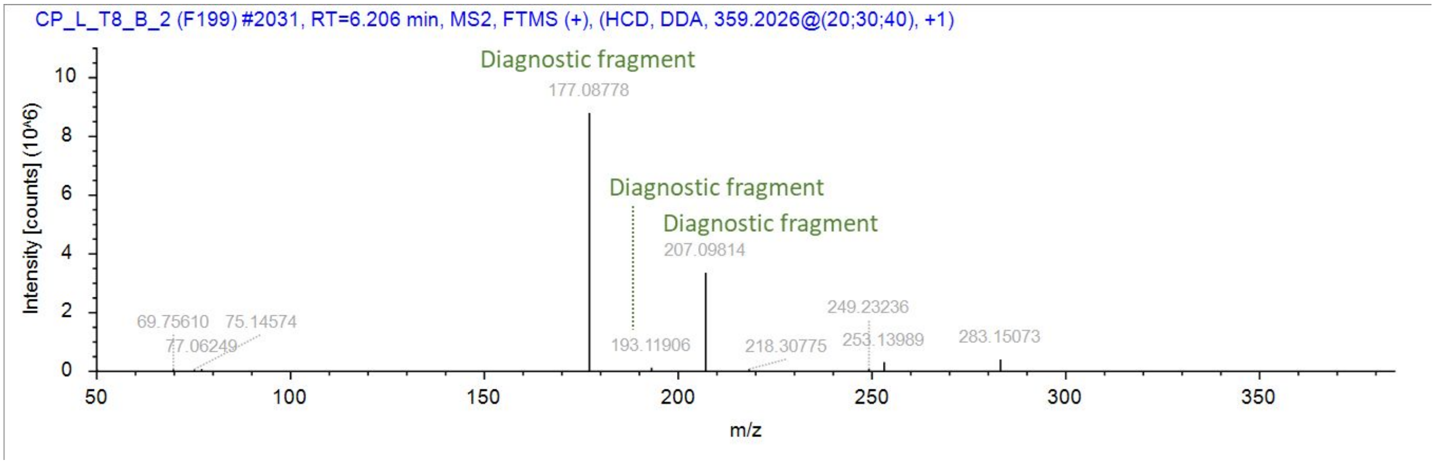

HMMM $TP_{HMMM}345$ MS1 Spectrum

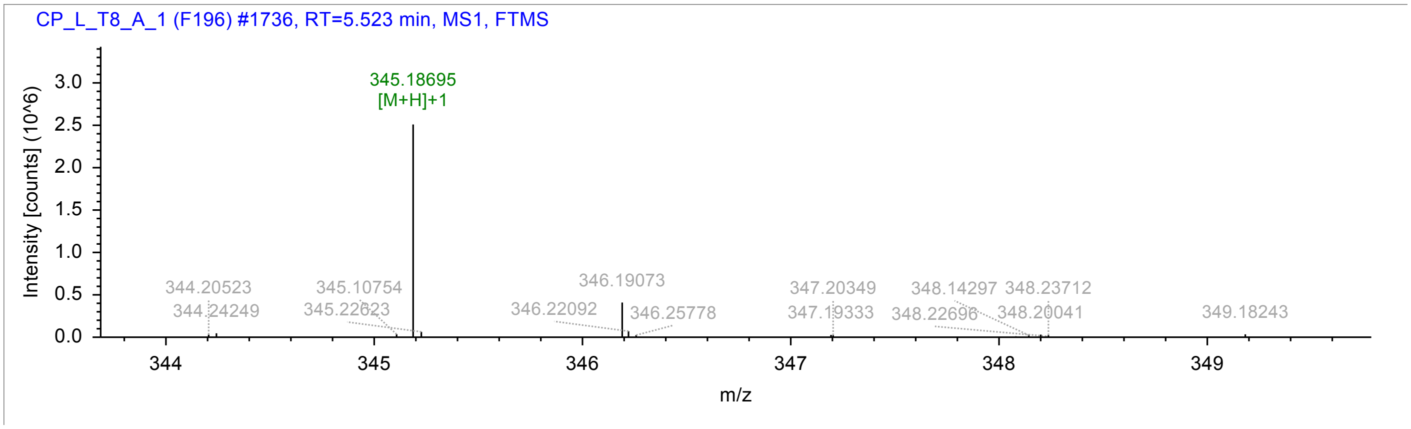

HMMM $TP_{HMMM}345$ MS2 Spectrum

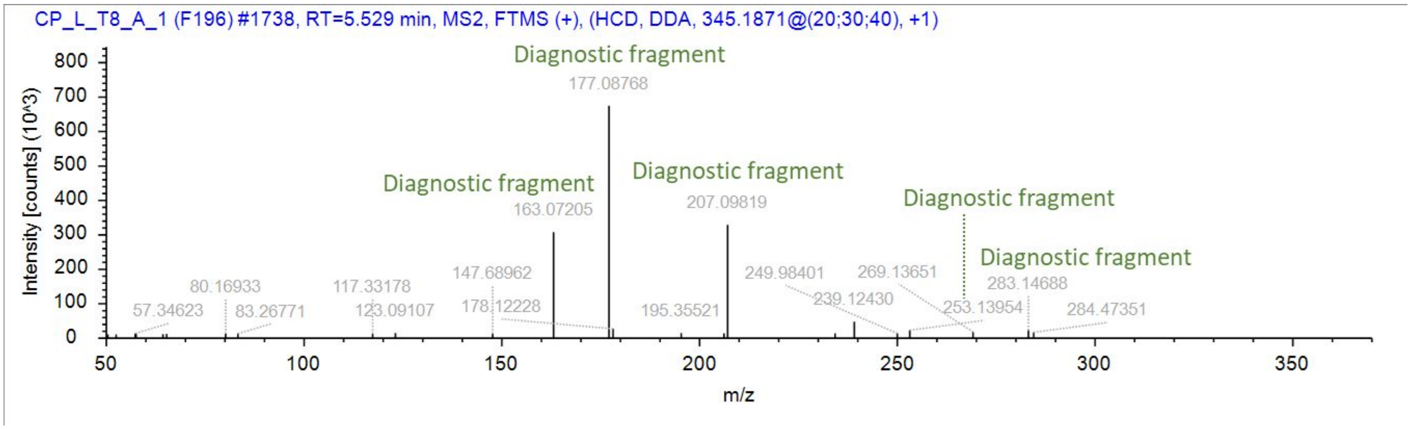

HMMM TP_{HMMM}345 isomer MS1 Spectrum

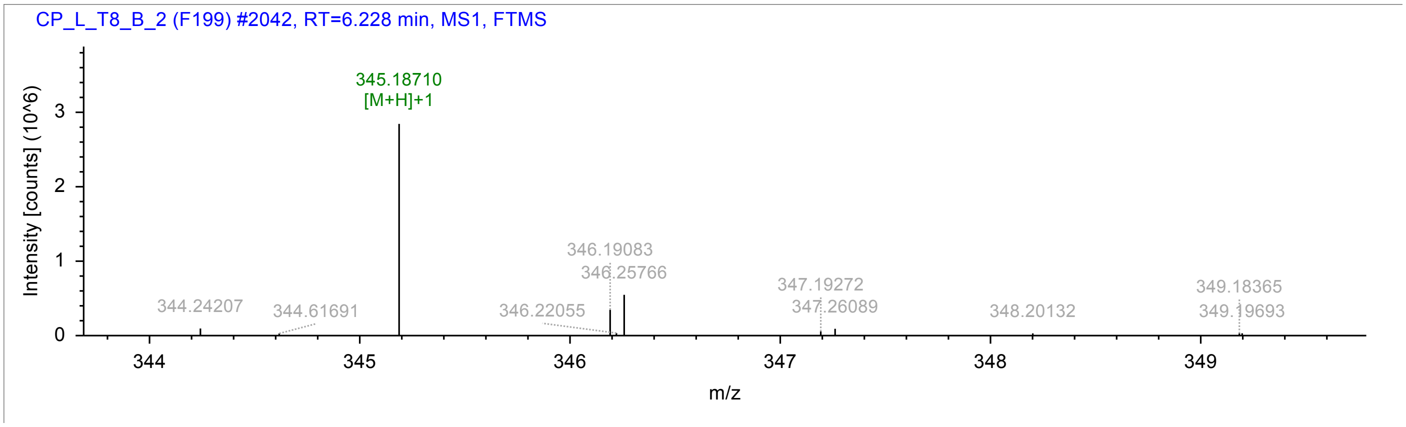

HMMM TP_{HMMM}345 isomer MS2 Spectrum

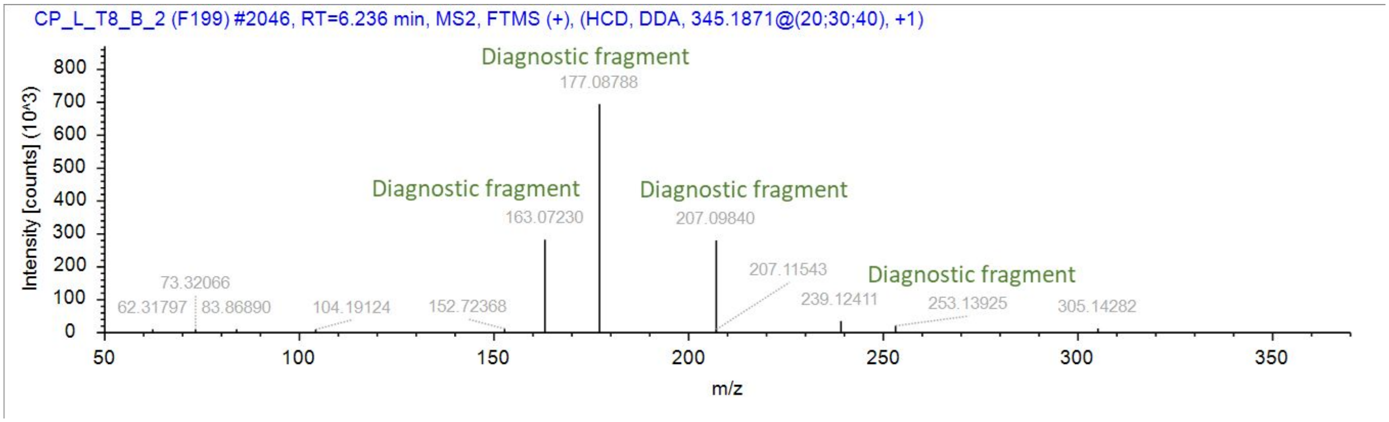

HMMM TP_{HMMM}331 MS1 Spectrum

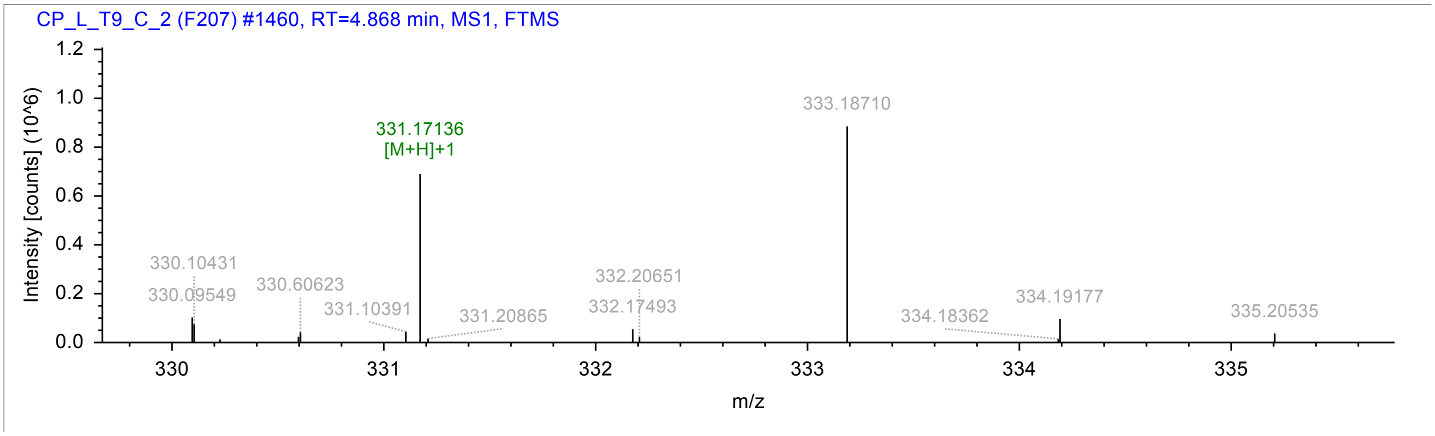

HMMM TP_{HMMM}331 MS2 Spectrum

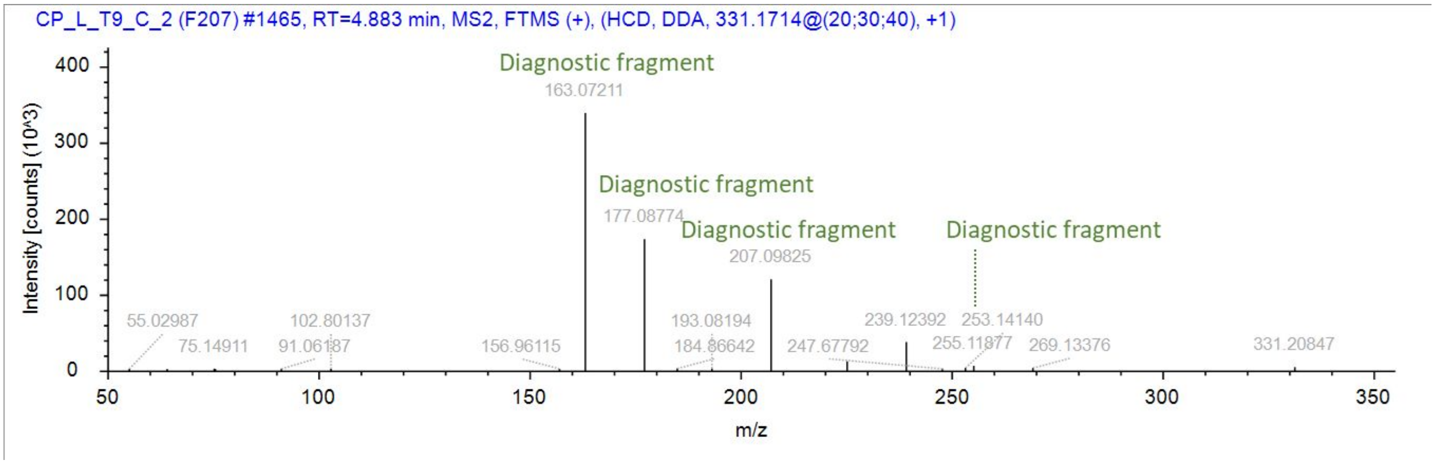

HMMM TP_{HMMM}301 MS1 Spectrum

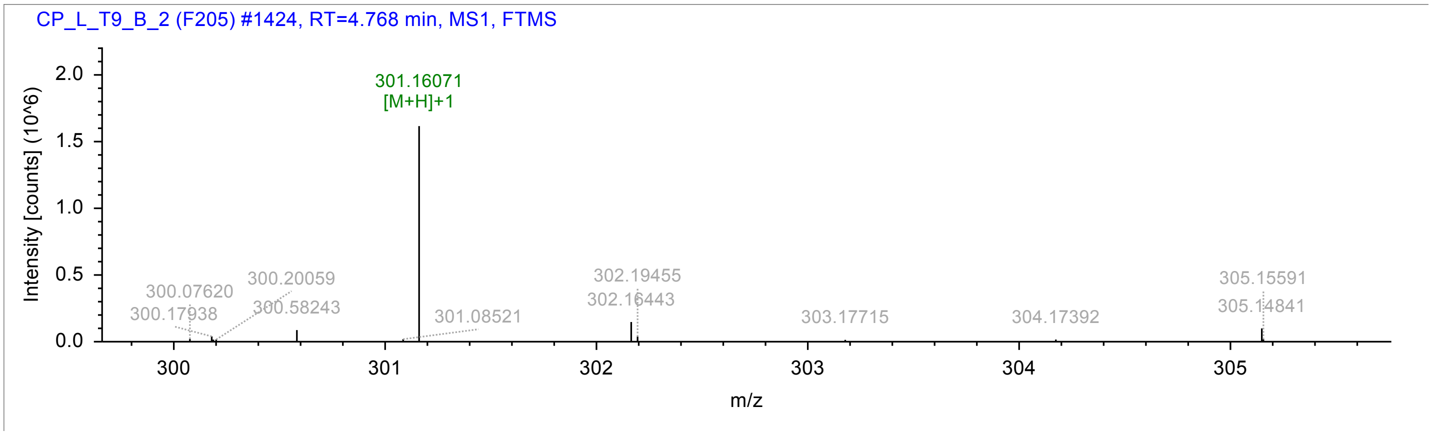

HMMM TP_{HMMM}301 MS2 Spectrum

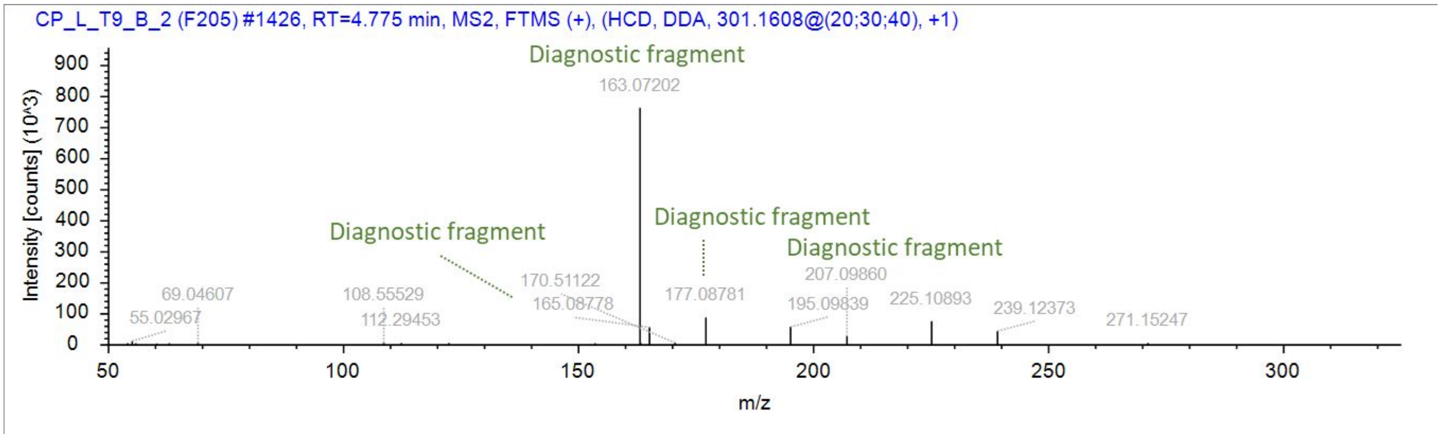

HMMM TP_{HMMM}303 MS1 Spectrum

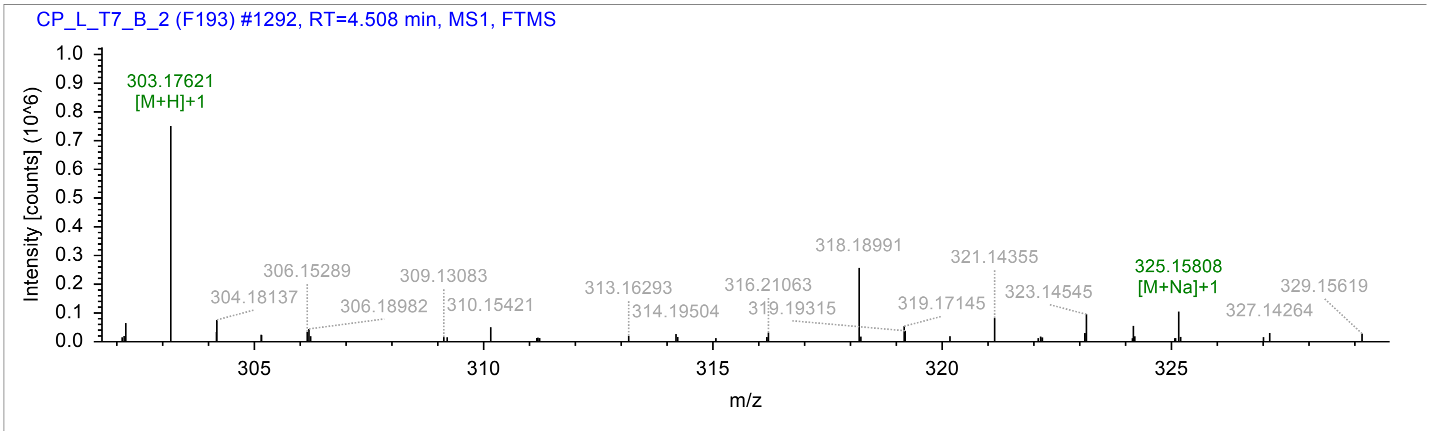

HMMM TP_{HMMM}303 MS2 Spectrum

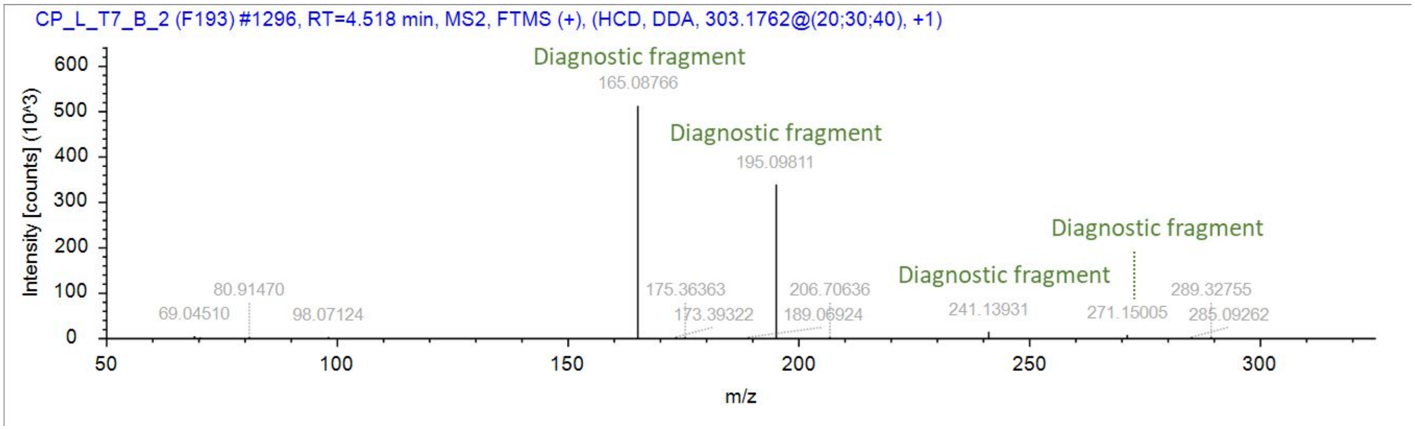

HMMM $TP_{HMMM}271$ MS1 Spectrum

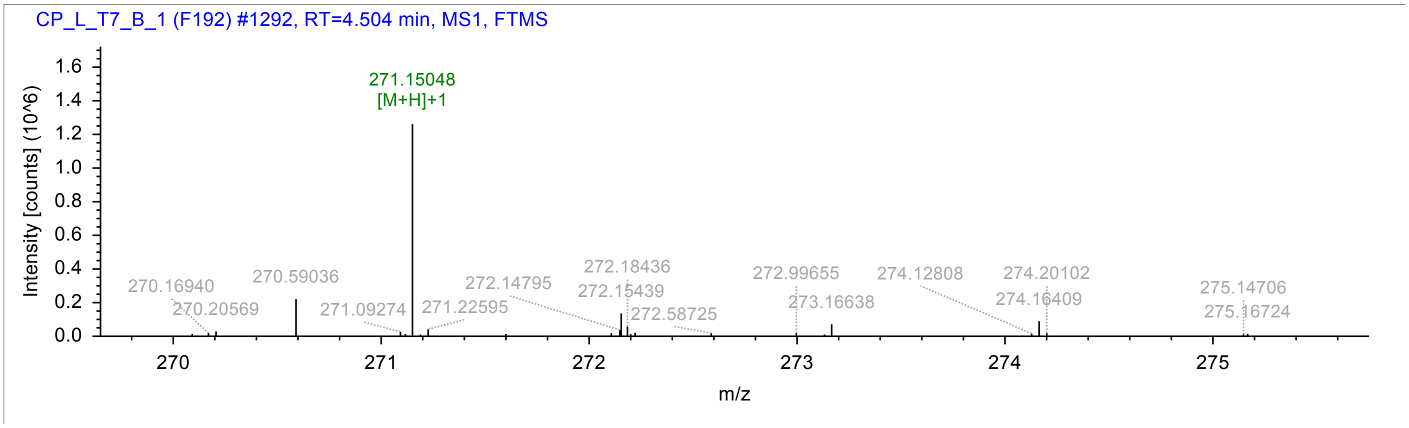

HMMM $TP_{HMMM}271$ MS2 Spectrum

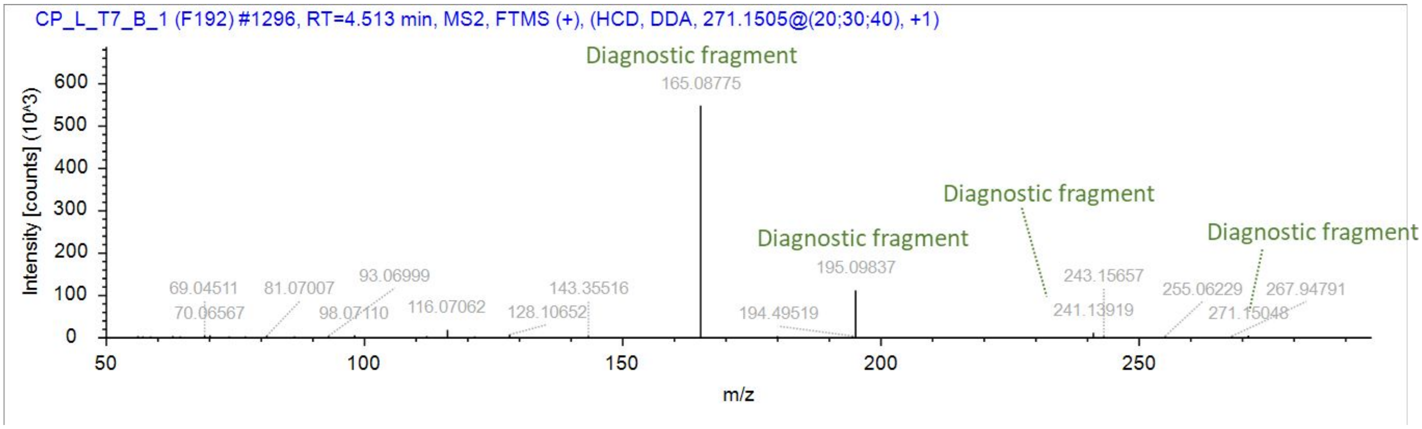

HMMM TP_{HMMM}546 MS1 Spectrum

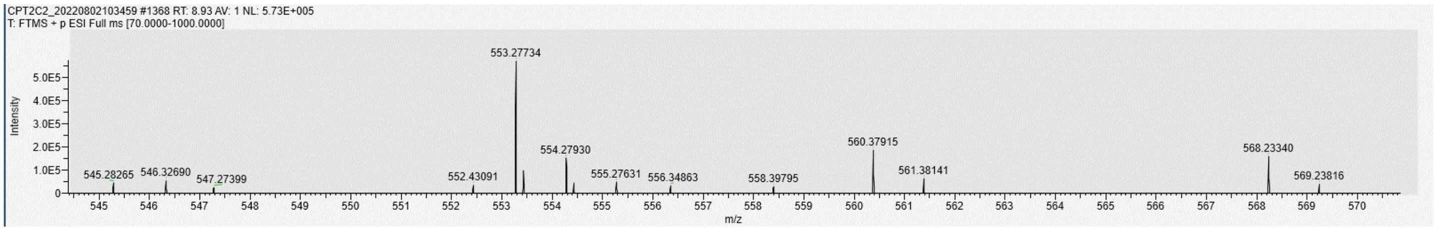

HMMM TP_{HMMM}546 MS2 Spectrum

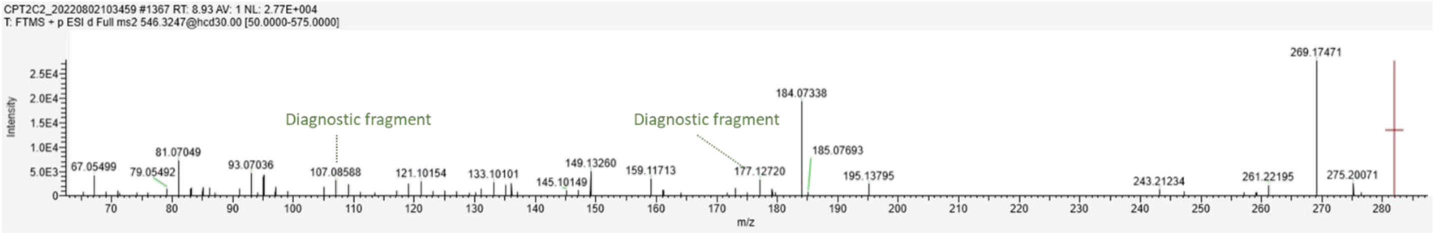

E) DPG

DPG MS1 Spectrum

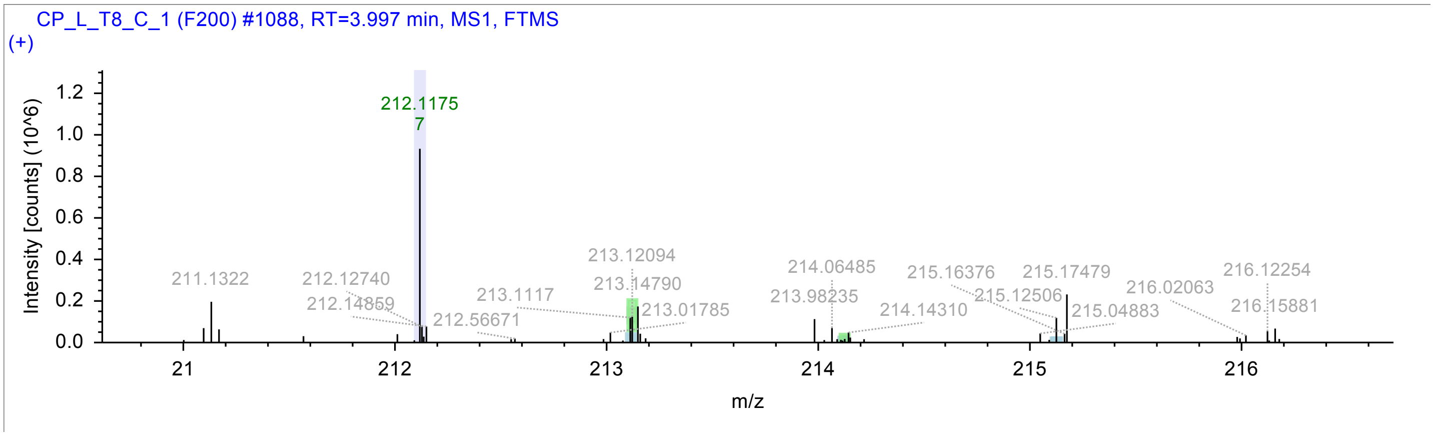

DPG MS2 Spectrum

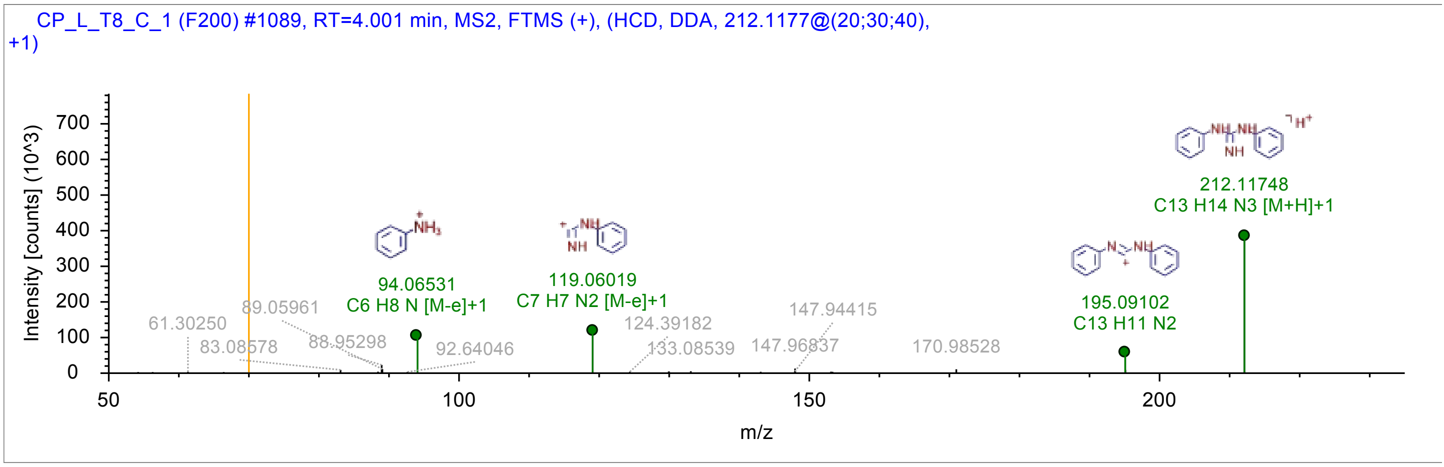

DPG TP$_{DPG}$266 MS1 Spectrum

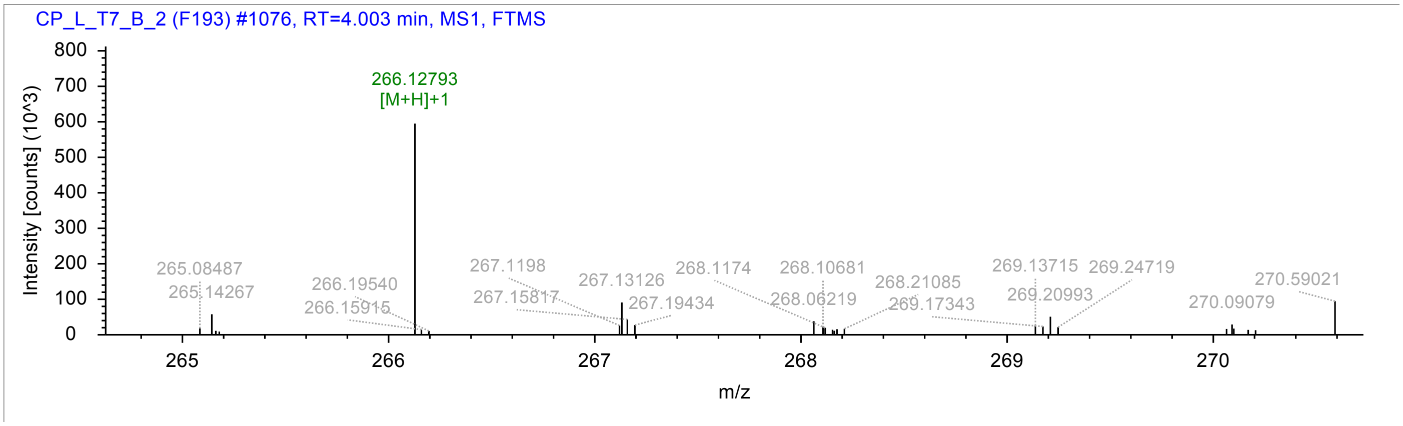

DPG TP$_{DPG}$266 MS2 Spectrum

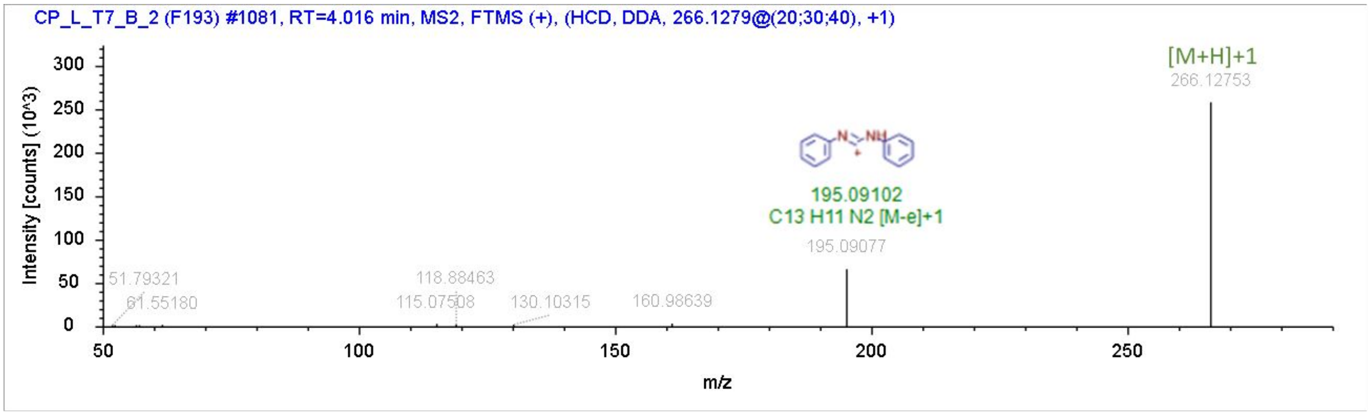

DPG TP_{DPG}268 MS1 Spectrum

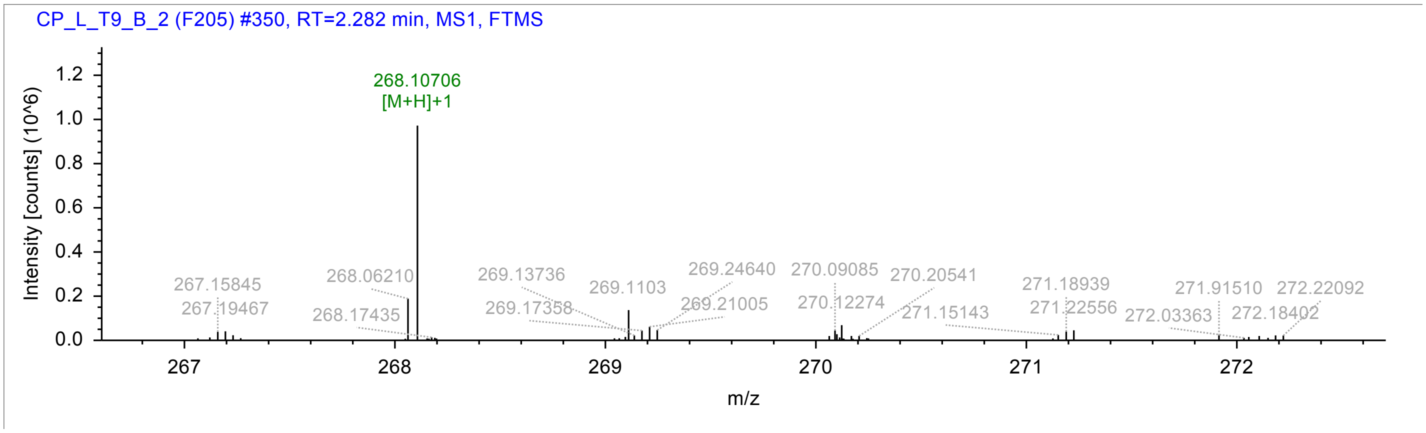

DPG TP_{DPG}268 MS2 Spectrum

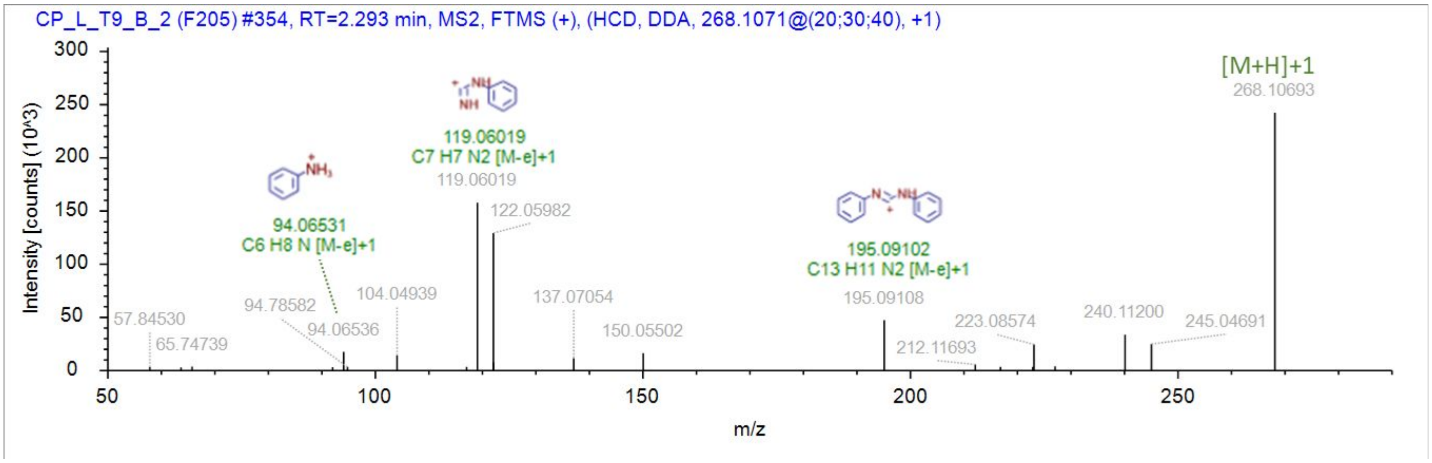

DPG TP_{DPG}270 MS1 Spectrum

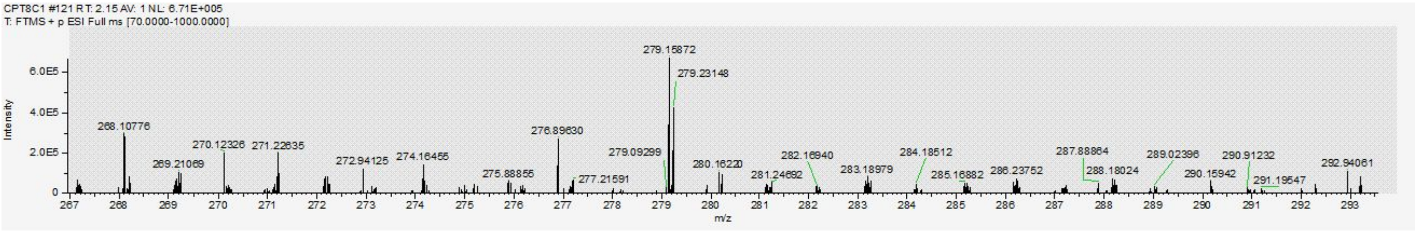

DPG TP_{DPG}270 MS2 Spectrum

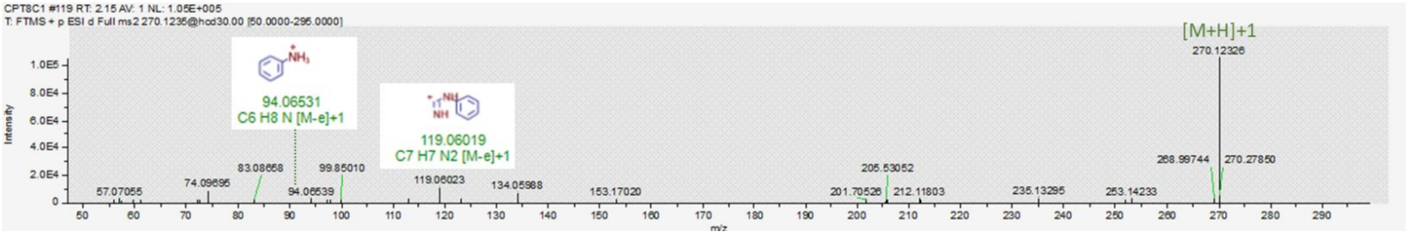

Figure S4: HRMS Spectra parent compounds and transformation products for A)BTZ, B)6PPD, C)6PPD-q, D)HMMM, and E)DPG.
